# Postural control and trunk muscle activation in transtibial amputees: A pilot electromyographic study

**DOI:** 10.1371/journal.pone.0333213

**Published:** 2025-09-26

**Authors:** Hüseyin Çelik, Mustafa Cem Türkmen, Ali İmran Yalçın, Semra Topuz

**Affiliations:** 1 Deparment of Therapy and Rehabilitation, Physiotherapy Program, Sungurlu Vocational School, Hitit University, Corum, Turkey; 2 Deparment of Therapy and Rehabilitation, Disabled Care and Rehabilitation Program, Sabuncuoglu Serefeddin Health Services Vocational School, Amasya University, Amasya, Turkey; 3 Movement Analysis Laboratory, Faculty of Physical Therapy and Rehabilitation, Hacettepe University, Ankara, Turkey; Roma Tre University: Universita degli Studi Roma Tre, ITALY

## Abstract

Transtibial amputation affects postural stability, requiring trunk muscle analysis to support balance and rehabilitation. This pilot study aimed to compare trunk muscle activations during postural sway and limits of stability in unilateral transtibial amputees with healthy controls and investigate the relationship between postural control and trunk muscle activations in transtibial amputees. Accordingly, it was hypothesised that transtibial amputees would exhibit altered postural control and trunk muscle activation, both compared to controls and between limbs. This preliminary observational cross-sectional study included a transtibial amputee group (n = 10) and a healthy control group (n = 10). Static and dynamic balance were assessed using a Bertec force platform. Trunk muscle activation was measured bilaterally using the Delsys wireless surface electromyography system while balance was assessed. Subsequently, group differences in postural control and trunk muscle activation were analyzed. Transtibial amputee group showed significantly greater limits of stability distance on the amputated side compared to controls’ non-dominant side (p < 0.05). Additionally, transtibial amputee group exhibited greater lateral postural sway under compliant surface conditions compared to healthy controls (p < 0.05). During limits of stability assesment, external oblique activation was higher on the intact side of transtibial amputee group compared to controls’ dominant side and higher than on the amputated side (p < 0.05). Multifidus activation on the amputated side during compliant surface with eyes open was greater than the intact side (p < 0.05). Longissimus dorsi activation on the intact side in amputee group exceeded controls’ dominant side under all conditions (p < 0.05). Moreover, longissimus dorsi activation on the amputated side was significantly higher than on the controls’ nondominant side during compliant surface with eyes closed (p < 0.05). Preliminary findings highlight disrupted postural control and trunk muscle activation in transtibial amputees, indicating the need for targeted rehabilitation and larger studies.

## 1. Introduction

Postural stability is a fundamental component of independent daily living [1–3]. During upright standing, the postural control system collects inputs from the visual, vestibular, and somatosensory systems and aims to use these inputs position the center of pressure within the support surface [4]. However, lower extremity amputation disrupts this system, potentially causing a loss of somatosensory input, decreased muscle activity, and reduced joint mobility. These alterations may lead to balance deficits and significant impairment of overall body function [5–9].

In transtibial amputation, the loss of the foot – an essential proprioceptive organ – necessitates the reprogramming of the postural control system. This adaptation process is influenced by factors such as weight-bearing asymmetries, somatosensory deficits, a reduced base of support, and increased joint stiffness, all of which contribute to the development of compensatory mechanisms [10–13]. These mechanisms have a significant impact on neuromuscular motor performance, particularly the postural sway observed during upright standing [11]. Although the use of prostheses offers a means of restoring lower extremity function, existing prosthetic technologies remain inadequate in providing the sensory feedback required for optimal postural control [4]. Prosthetic design and rehabilitation strategies significantly influence postural control mechanisms [14].

Individuals using unilateral lower extremity prostheses often demonstrate significant trunk adaptations in both the frontal and sagital planes [15,16]. In addition to the lower limb asymmetries caused by amputation, the asymmetric trunk movements observed in these individuals [15,17–19] may lead to differences in trunk muscle activation between the intact and amputated sides. Although numerous studies in the literature have addressed asymmetric postural and balance problems in amputees, the underlying mechanisms of these problems – particularly those involving trunk muscles – remain insufficiently explored [13,20–23]. Such asymmetries may predispose individuals to chronic neuromuscular conditions, including back and limb pain, vascular diseases, and early-onset arthritis [21,22].

Understanding alterations in trunk muscle activation following transtibial amputation is crucial for identifying novel and modifiable factors that can be addressed during the rehabilitation process. This study was designed as a pilot to provide preliminary data and to lay the groundwork for a larger-scale investigation. The aim of this study is to examine the effects of unilateral transtibial amputation on postural control and trunk muscle activation and to investigate the relationship between postural control and trunk muscle performance in amputees. To this end, this study hypothesised that the transtibial amputees would exhibit significant differences postural control and trunk muscle activation compared to the healthy individuals. Furthermore, it was hypothesised that trunk muscle activation would differ significantly between the amputated and intact sides in the transtibial amputees.

## 2. Materials and methods

### 2.1 Study design

This study was conducted at the Faculty of Physical Therapy and Rehabilitation, Hacettepe University. Ethical approval was granted by the University Non-Interventional Clinical Research Ethics Board (Approval number: GO21/1104). Written informed consent was obtained from all participants prior to their inclusion in the study.

### 2.2 Participants

The study included a transtibial amputee group (TA) and a healthy control group (CG). Inclusion criteria for the TA were participants aged 18–45 years who had undergone amputation for traumatic reasons, had been using a prosthesis for at least one year, and reported no stump pain, phantom sensation, or phantom pain at rest or during activity. Participants were also required to have an activity level of K3 or K4 according to the Medicare Functional Classification Level. The CG group consisted of healthy individuals with similar demographic characteristics to the TA, without any neurological or orthopaedic conditions. Individuals with unilateral transtibial amputation who visited prosthetic rehabilitation centres, orthopaedic clinics, or physical therapy clinics, met the inclusion criteria, and voluntarily agreed to participate were included in the study sample.

Exclusion criteria for both groups included individuals with limited hip, knee, or ankle range of motion; muscle contractures that impaired gait or activities of daily living; a history of falls within the past year; a body mass index (BMI) >30 kg/m²; or the need for assistive devices to stand and maintain balance.

### 2.3 Instrumentation and experimental protocol

After written consent was obtained, demographic and physical characteristics of the participants were recorded. For the TA, additional data related to amputation and prosthesis use were documented, including the cause and side of amputation, time since amputation, lower limb length, stump length, daily prosthesis usage duration, and details of prosthetic components (socket design, foot type, and suspension system). For amputee BMI calculations, 6.5% of the total body weight was added to compensate for the loss of body mass resulting from limb amputation [24]. Authors had access to information that could identify individual participants during or after data collection.

Participants were recruited to the study between January 2023 and June 2023. All assessments were conducted in a quiet environment free from potential distractions. None of the participants reported any pain (e.g., lower back pain or stump pain) during the tests.

#### 2.3.1 Postural control.

Postural control assessments were conducted using the Bertec Balance Check Screener™ BP5046 force platform (Bertec BP5046 balance plate platform; Bertec, Corp., Columbus, OH, USA). This force platform objectively measures vertical force and Center of Pressure (CoP) changes. A foam pad of identical dimensions to the platform was used for soft surface assessments. Participants were instructed to place their feet symmetrically along the midline of the platform or foam pad in a self-selected comfortable position.

During static postural assessments, participants were asked to maintain balance and remain as still as possible in four conditions: eyes open on a firm surface (normal surface eyes open (NSEO)), eyes closed on a firm surface (normal surface eyes closed (NSEC)), eyes open on a foam surface (compliant surface eyes open (CSEO)), and eyes closed on a foam surface (compliant surface eyes closed (CSEC)). Antero-posterior and lateral postural sway parameters were recorded as indicators of static balance.

For the Limits of Stability (LoS) assessment, which provides insight into dynamic balance, foot positioning was identical to that used for the postural sway assessments. LoS was calculated by measuring the maximum voluntary displacement of the CoP in each of the four directions using the Bertec balance platform. Participants were instructed to move their body (but without bending the body) as far forward, backward, left, and right as possible without lifting their feet off the platform. The average of three trials was taken for each direction, and the total LoS distance was computed. A higher LoS value indicates better dynamic balance control. Measurements were conducted with participants wearing their usual footwear, as the position of the prosthetic foot is adjusted based on the shoes worn by the individual in daily life. In addition, all assessments were repeated three times.

Postural sway and LoS data were recorded as CoP displacements in the frontal plane. For the TA, data were categorized as intact side (IS) and amputated side (AS), whereas for the CG, data were recorded as dominant side (DS) and non-dominant side (NDS). The IS in the TA was matched to the DS in the CG, and the AS in the TA was matched to the NDS in the CG for between-group comparisons. This selection was based on the functional similarity of the dominant role of the intact side in maintaining balance to the dominant side of healthy individuals, and the more passive, supportive role of the amputated side to the non-dominant side. Similar matching strategies have also been employed in studies investigating lateral asymmetry and balance mechanisms in the literature [25–27]. The participants’ starting side was chosen at random during the trials. The average of three trials was calculated for each parameter of the LoS assessment and postural sway, and these mean values were used in the analyses. A lower postural sway range indicates better balance performance in participants, whereas a higher LoS value also reflects improved balance performance in participants [28].

#### 2.3.2 Trunk muscle activation.

Trunk muscle activation was assessed using the Delsys Trigno IM wireless surface electromyography (sEMG) system (Delsys Inc., Natick, Massachusetts, USA) [29,30]. Bilateral measurements of the muscles longissimus dorsi (LD), multifidus (MF), rectus abdominis (RA), and obliquus externus (EO) were obtained using an eight-channel wireless system equipped with 99.9% Ag surface bar electrodes with a fixed 1 cm inter-electrode distance and a portable data acquisition system. sEMG signals were recorded with a common mode rejection ratio (CMRR) of <80 dB and a sampling rate of 1926 Hz, using the wireless multi-channel sEMG acquisition system.

The preparation phase involved shaving the skin at the electrode placement sites and cleansing the area with 70% isopropyl alcohol to minimize skin impedance. Electrodes were securely atached to the skin surface using strong double-sided adhesive tape. Although cross-talk is a known limitation of sEMG systems, electrode placement in this study was performed in accordance with the SENIAM (Surface Electromyography for the Non-Invasive Assessment of Muscles) guidelines [31,32], aiming to minimize cross-talk and ensure standardized electrode placement across all participants. sEMG recordings were performed simultaneously during both the LoS and postural sway assessments.

To normalize the EMG signals obtained from the muscles, the maximum voluntary isometric contraction (MVIC) of each muscle was measured in three trials, with one-minute rest intervals between repetitions [33,34]. The raw data were analyzed using the Delsys EMG Works Analysis software. Subsequently, to optimise the signal quality and to remove power line noise, a 50 Hz notch filter and filter parameters were set to 4th order butterworth, band pass filter, cut off frequencies 20–450 Hz, Root Mean Square (RMS) window length 100 ms, window overlap 50 ms.

The analysis process was completed in four stages:

1. The MVIC data of the assessed muscles and the EMG data recorded during the balance assessment were filtered.
2. RMS values of the filtered MVIC data were computed, and the highest MVIC value among the three trials (MVIC) was selected.
3. The EMG data obtained during the balance assesment and then filtered was applied amplitude analysis.
4. The arithmetic mean of the amplitude analysis values from the three trials was calculated as %MVIC, and the results were recorded.

### 2.4 Statistical analysis

Statistical analyses were conducted using IBM SPSS Statistics 26.0 (SPSS Inc., Chicago, IL, USA). Descriptive statistics were presented as frequencies and percentages for categorical variables and as medians and percentiles for numerical variables. The normal distribution analysis of the data was examined using visual and analytical methods (Shapiro-Wilk test). Because all numeric variables showed non-normal distribution, non-parametric tests were used. The Mann-Whitney U Test was used to compare numerical variables between TA and CG [35]. Within TA, numerical variables between the AS and IS were compared using the Wilcoxon Signed-Rank Test.

Relationships between numerical variables were assessed using Spearman’s correlation test. Correlation coefficient values were interpreted as follows: 0.00–0.20 (very weak), 0.21–0.40 (weak), 0.41–0.60 (moderate), 0.61–0.80 (strong), and 0.81–1.00 (very strong) [35]. A type 1 error level of less than 5% (p < 0.05) was considered statistically significant. Given the pilot nature of the study and the limited sample size, these statistical results are presented for preliminary evaluation and should be interpreted with caution.

## 3. Results

A total of 32 individuals were initially evaluated for inclusion in the study (15 unilateral transtibial amputees and 17 healthy controls). All participants underwent eligibility screening based on the predetermined inclusion and exclusion criteria. Following the screening process, 20 participants (10 TA and 10 CG) met the eligibility criteria and were included in the study. A total of 12 individuals were excluded from the study for not meeting the inclusion criteria: five amputees due to BMI > 30 kg/m² (n = 1), limited range of motion (ROM) (n = 2), and low back pain (n = 2); and seven controls due to BMI > 30 kg/m² (n = 3) and neurological or orthopedic conditions (n = 4). Given the pilot nature of this study, the analyses were conducted on this limited sample and are presented as preliminary findings intended to inform future larger-scale investigations.

**Fig 1:**
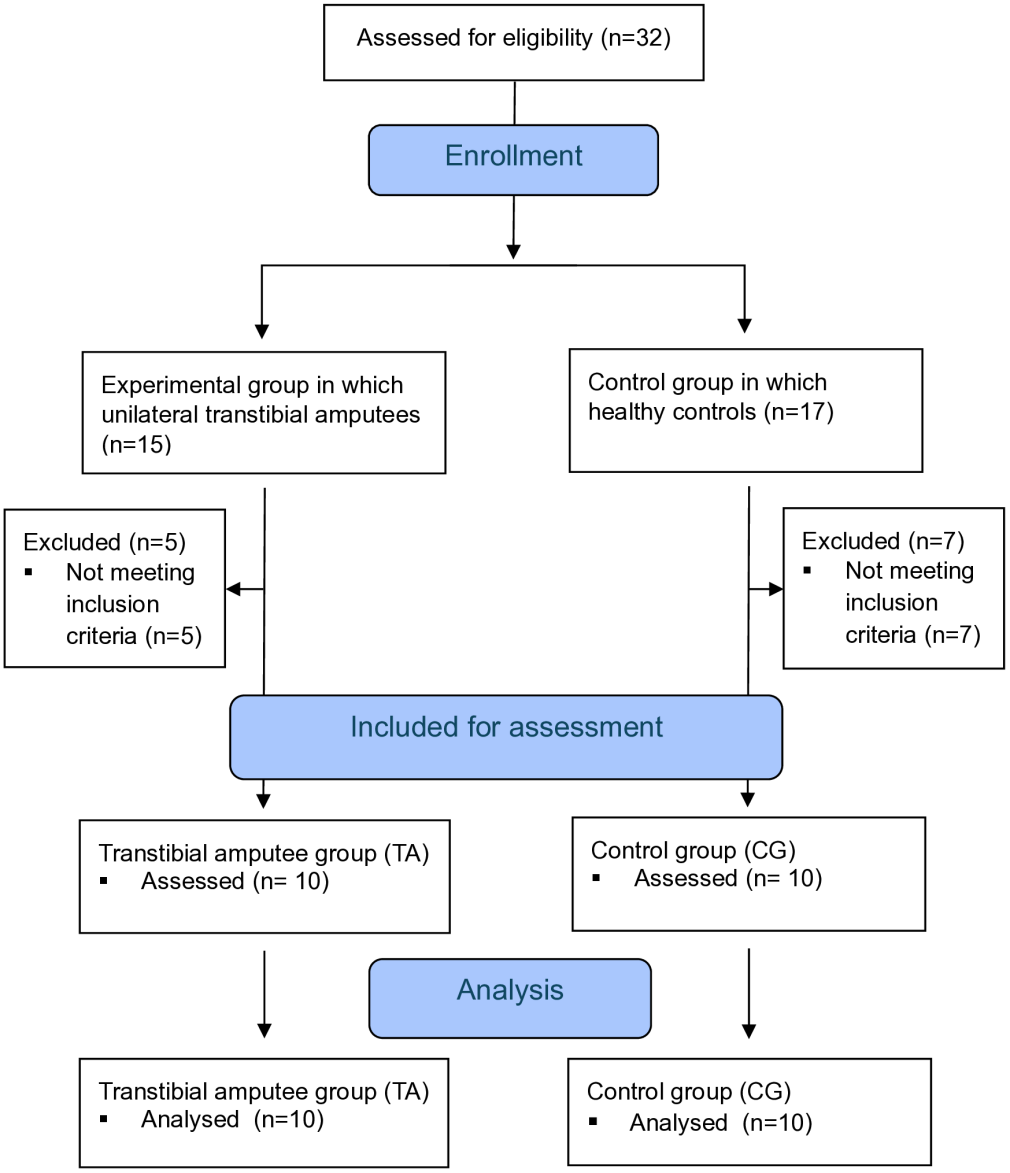
Participant flowchart diagram.

All 20 enrolled participants successfully completed the study, including postural control and electromyographic (EMG) assessments. No participants withdrew or were lost to follow-up during the study period. For data analysis, since there were no missing data or losses to follow-up, all 20 participants (10 TA and 10 CG) were included in the final statistical analyses (Fig 1).

Groups were statistically similar in terms of age, weight, height, BMI and lower limb length on the IS/DS (p > 0.05). The stump length, age at amputation, time since amputation, and duration of prosthesis use for the TA are shown in Table 1 (Table 1). Among the individuals in the TA, 7 had right-sided amputations, and 3 had left-sided amputations, with all participants using total contact sockets. Suspension was achieved through active vacuum in 8 participants and passive vacuum in 2 participants. All participants used carbon prosthetic feet (Table 1).

**Table 1:** Demographic and amputation-specific characteristics of the participants.

Variables		Amputee Group (n = 10)	Control Group (n = 10)	p
		Median (IQR) (25-75 Persantil)	Median (IQR) (25-75 Persantil)	
Age (year)		35.5 (31-41)	34.5 (29-37)	0.472
Weight (kg)		76.35 (72.8-87.6)	70.45 (66.95-83.35)	0.173
Height (m)		1.78 (1.74-1.8)	1.72 (1.71-1.76)	0.063
Body Mass Index (BMI) (kg/m²)		24.26 (22.94-27.84)	23.85 (22.38-27.68)	0.705
Lower limb length (cm)		85 (71-93)	87 (85-92)	0.570
Stump length (cm)		16.75 (16-18)		
Age at amputation (year)		21 (20-25)		
Time since amputation (year)		15 (5-19)		
Duration of prosthesis use (year)		15.12 (12-19)		
		n (%)		
Amputated side	Right	7 (70)		
	Left	3 (30)		
Prosthetic suspension system	Passive vacuum	2 (20)		
	Active vacuum	8 (80)		
Prosthetic foot	Carbon	10 (100)		

Mann-Whitney U-test.

n, number of individuals; %, percentage; (cm), centimeter; (m), meter; (kg), kilogram.

No significant differences were found between the TA and CG in LoS anterior, posterior, and IS/DS values. However, the LoS distance of the NDS in CG was found to be lower than the LoS distance of the AS in TA, and this difference was statistically significant (p = 0.019). In static stance, lateral sway was significantly higher in the TA under the CSEO (p = 0.023) and CSEC (p = 0.002) conditions compared to the CG. No differences were observed between the groups in other postural sway ranges (p > 0.05) (Table 2).

**Table 2:** Inter-group differences in postural control measurements.

*Variables*		Amputee Group (n = 10)	Control Group (n = 10)	p
**LoS (cm)**	**Anterior**	7.006 (6.134-8.15)	8.688 (6.51-10.191)	0.190
	**Posterior**	5.965 (4.088-7.308)	4.246 (3.833-6.714)	0.579
	**IS/DS**	9.765 (8.416-13.039)	9.239 (6.356-9.844)	0.165
	**AS/NDS**	11.802 (10.682-15.174)	10.313 (8.116-11.369)	0.019*
**Antero-posterior sway (cm)**	**NSEO**	0.45 (0.434-0.548)	0.516 (0.446-0.601)	0.579
	**NSEC**	0.873 (0.67-0.985)	0.736 (0.551-0.859)	0.280
	**CSEO**	0.597 (0.464-0.646)	0.544 (0.433-0.628)	0.631
	**CSEC**	1.067 (1.051-1.433)	0.912 (0.756-1.09)	0.089
**Lateral sway (cm)**	**NSEO**	0.202 (0.19-0.258)	0.164 (0.15-0.194)	0.105
	**NSEC**	0.298 (0.265-0.378)	0.23 (0.151-0.283)	0.113
	**CSEO**	0.458 (0.407-0.561)	0.308 (0.272-0.414)	0.023*
	**CSEC**	0.732 (0.623-0.799)	0.453 (0.34-0.495)	0.002*

*p < 0.05 was considered statistically significant; Mann-Whitney U-test. Values are presented as median (25th/75th percentile).

LoS, limits of stability; NSEO, normal surface eyes open; NSEC, normal surface eyes closed; CSEO, compliant surface eyes open; CSEC, compliant surface eyes closed; (cm), centimeter; AS, amputated side; IS, intact side; DS, dominant side; NDS, nondominant side.

During the LoS assessment, EMG amplitude levels of the all muscles were found to be increased in the TA. Notably, this increase was statistically significant for the EO on the IS/DS when compared between groups (p = 0.023). Analysis of EMG amplitude levels during postural sway in static stance revealed that all measured muscles in the TA had higher EMG amplitudes than those in the CG group. Statistically significant differences were observed in the EMG amplitude levels of the LD on the IS/DS in the NSEC (p = 0.007), NSEO (p = 0.035) and CSEO (p = 0.015) conditions, and in both sides of the LD in the CSEC (p = 0.023, p = 0.005) condition. No other significant differences were found between the groups (p > 0.05) (Table 3).

**Table 3:** Inter-group differences in trunk muscle activation measurements.

*Variables*			Amputee Group (n = 10) Median (IQR)	Control Group (n = 10) Median (IQR)	p
**LoS**	**Longissimus Dorsi**	**AS/NDS**	14.26 (7.06-17.93)	7.14 (4.95-13.52)	0.052
		**IS/DS**	13.66 (7.59-14.11)	6.56 (3.96-14.48)	0.143
	**Multifidus**	**AS/NDS**	16.44 (12.46-22.47)	12.84 (9.98-19.76)	0.315
		**IS/DS**	15.22 (13.54-16.91)	12.45 (7.49-19.41)	0.247
	**Rectus Abdominis**	**AS/NDS**	3.84 (2.58-4.65)	2.4 (1.42-3.44)	0.165
		**IS/DS**	4.76 (2.04-7.19)	2.38 (1.01-2.98)	0.105
	**External Oblique**	**AS/NDS**	11.1 (5.93-11.51)	6.08 (5.23-8.77)	0.052
		**IS/DS**	13.02 (9.18-20.76)	6.54 (5.9-7.61)	0.023*
**NSEC**	**Longissimus Dorsi**	**AS/NDS**	5.4 (4.4-9.35)	4.55 (2.44-6.18)	0.353
		**IS/DS**	6.23 (3.83-7.44)	2.94 (2.51-3.31)	0.007*
	**Multifidus**	**AS/NDS**	6.75 (4.1-16.13)	5.24 (3.92-6.8)	0.436
		**IS/DS**	11.46 (6.58-12.12)	4.79 (4.15-8.32)	0.075
	**Rectus Abdominis**	**AS/NDS**	2.49 (1.28-5.87)	1.97 (1.27-2.88)	0.353
		**IS/DS**	2.92 (1.45-6.56)	1.79 (0.87-2.1)	0.143
	**External Oblique**	**AS/NDS**	5.16 (4.08-6.34)	4.82 (3.29-5.62)	0.579
		**IS/DS**	8.88 (2.68-12.1)	5.45 (4.23-6.07)	0.247
**NSEO**	**Longissimus Dorsi**	**AS/NDS**	5.14 (2.8-7.39)	4.06 (2.29-5.53)	0.393
		**IS/DS**	4.81 (3.25-6.97)	2.69 (1.81-2.96)	0.035*
	**Multifidus**	**AS/NDS**	7.06 (4.85-17.05)	5.67 (3.69-6.47)	0.247
		**IS/DS**	7.1 (4.03-11.2)	5.27 (2.85-7.37)	0.315
	**Rectus Abdominis**	**AS/NDS**	2.41 (1.22-4.84)	1.7 (1.12-2.72)	0.393
		**IS/DS**	2.88 (1.34-6.46)	1.8 (0.87-2.18)	0.089
	**External Oblique**	**AS/NDS**	5.44 (3.77-6.8)	4.81 (3.53-5.23)	0.796
		**IS/DS**	8.66 (2.7-14.86)	5.44 (3.39-5.81)	0.353
**CSEC**	**Longissimus Dorsi**	**AS/NDS**	6.31 (5.04-9.87)	3.59 (3.03-5.17)	0.023*
		**IS/DS**	4.96 (4.37-8.11)	2.7 (2.38-3.74)	0.005*
	**Multifidus**	**AS/NDS**	11.69 (4.03-16.18)	6.58 (5.11-8.15)	0.123
		**IS/DS**	8.29 (6.24-10.77)	6.11 (2.8-8)	0.123
	**Rectus Abdominis**	**AS/NDS**	3.1 (1.32-5.59)	1.86 (1.05-2.89)	0.190
		**IS/DS**	2.69 (1.45-5.02)	1.81 (0.83-2.2)	0.105
	**External Oblique**	**AS/NDS**	5.62 (4.54-7.4)	4.71 (3.34-5.87)	0.481
		**IS/DS**	8.12 (2.71-11.91)	4.46 (3.12-5.83)	0.165
**CSEO**	**Longissimus Dorsi**	**AS/NDS**	4.84 (4.24-8.07)	3.24 (2.33-4.95)	0.105
		**IS/DS**	6.05 (3.42-8.03)	2.83 (2.03-3.3)	0.015*
	**Multifidus**	**AS/NDS**	11.43 (3.91-18.56)	7.02 (3.23-8.11)	0.063
		**IS/DS**	7.68 (6.12-12)	4.56 (2.93-6.55)	0.089
	**Rectus Abdominis**	**AS/NDS**	2.34 (1.21-4.85)	2.16 (1.27-2.96)	0.529
		**IS/DS**	2.62 (1.41-5.06)	1.9 (0.88-2.1)	0.165
	**External Oblique**	**AS/NDS**	5.62 (3.87-6.59)	4.35 (3.85-4.99)	0.353
		**IS/DS**	7.96 (2.83-12.16)	5.05 (3.65-5.95)	0.165

*p < 0.05 was considered statistically significant; Mann-Whitney U-test. Values are presented as median (25th/75th percentile).

LoS, limits of stability; NSEO, normal surface eyes open; NSEC, normal surface eyes closed; CSEO, compliant surface eyes open; CSEC, compliant surface eyes closed; AS, amputated side; IS, intact side; DS, dominant side; NDS, nondominant side.

When the EMG amplitude levels of the trunk muscles on the AS and IS were compared in the TA during LoS and postural sway, the activation of the EO on the AS during LoS was found to be significantly lower than that on the IS (p = 0.037). In static stance, the activation of the MF on the AS during CSEO was determined to be significantly higher than that on the IS (p = 0.022) (Table 4).

**Table 4:** Within-group comparisons of trunk muscle activation measurements in the amputee group.

*Variables*			Median (IQR)	p
**LOS**	**Longissimus Dorsi**	**AS**	14.26 (7.06-17.93)	0.508
		**IS**	13.66 (7.59-14.11)	
	**Multifidus**	**AS**	16.44 (12.46-22.47)	0.285
		**IS**	15.22 (13.54-16.91)	
	**Rectus Abdominis**	**AS**	3.84 (2.58-4.65)	0.799
		**IS**	4.76 (2.04-7.19)	
	**External Oblique**	**AS**	11.1 (5.93-11.51)	0.037*
		**IS**	13.02 (9.18-20.76)	
**NSEC**	**Longissimus Dorsi**	**AS**	5.4 (4.4-9.35)	0.878
		**IS**	6.23 (3.83-7.44)	
	**Multifidus**	**AS**	6.75 (4.1-16.13)	0.878
		**IS**	11.46 (6.58-12.12)	
	**Rectus Abdominis**	**AS**	2.49 (1.28-5.87)	0.878
		**IS**	2.92 (1.45-6.56)	
	**External Oblique**	**AS**	5.16 (4.08-6.34)	0.169
		**IS**	8.88 (2.68-12.1)	
**NSEO**	**Longissimus Dorsi**	**AS**	5.14 (2.8-7.39)	0.646
		**IS**	4.81 (3.25-6.97)	
	**Multifidus**	**AS**	7.06 (4.85-17.05)	0.114
		**IS**	7.1 (4.03-11.2)	
	**Rectus Abdominis**	**AS**	2.41 (1.22-4.84)	0.359
		**IS**	2.88 (1.34-6.46)	
	**External Oblique**	**AS**	5.44 (3.77-6.8)	0.059
		**IS**	8.66 (2.7-14.86)	
**CSEC**	**Longissimus Dorsi**	**AS**	6.31 (5.04-9.87)	0.386
		**IS**	4.96 (4.37-8.11)	
	**Multifidus**	**AS**	11.69 (4.03-16.18)	0.114
		**IS**	8.29 (6.24-10.77)	
	**Rectus Abdominis**	**AS**	3.1 (1.32-5.59)	0.721
		**IS**	2.69 (1.45-5.02)	
	**External Oblique**	**AS**	5.62 (4.54-7.4)	0.114
		**IS**	8.12 (2.71-11.91)	
**CSEO**	**Longissimus Dorsi**	**AS**	4.84 (4.24-8.07)	0.508
		**IS**	6.05 (3.42-8.03)	
	**Multifidus**	**AS**	11.43 (3.91-18.56)	0.022*
		**IS**	7.68 (6.12-12)	
	**Rectus Abdominis**	**AS**	2.34 (1.21-4.85)	0.799
		**IS**	2.62 (1.41-5.06)	
	**External Oblique**	**AS**	5.62 (3.87-6.59)	0.074
		**IS**	7.96 (2.83-12.16)	

*p < 0.05 was considered statistically significant; Wilcoxon signed-rank Test.

LoS, limits of stability; NSEO, normal surface eyes open; NSEC, normal surface eyes closed; CSEO, compliant surface eyes open; CSEC, compliant surface eyes closed; AS, amputated side; IS, intact side; DS, dominant side; NDS, nondominant side.

## 4. Discussion

This study aimed to investigate postural control and the trunk muscular mechanisms involved in postural control following unilateral transtibial amputation. The results indicate changes in postural control, adaptations in trunk muscles, and the effects of these adaptations on balance control after transtibial amputation.

Studies have demonstrated a positive correlation between lower limb length and balance performance, indicating that longer leg length is associated with better performance in dynamic balance conditions [36]. Additionally, BMI has been shown to influence balance, with higher BMI levels associated with greater balance impairment [37–39]. Beyond these factors, balance tends to decline with age, which has been identified as the strongest determinant of balance performance [40]. Considering these factors, which may influence balance and muscle activation outcomes, is particularly important in studies involving populations at risk for balance disorders. Accordingly, in our study, the similarity between the TA and CG in terms of age, sex, BMI and lower limb length (on the IS/DS) was crucial for isolating amputation-specific changes.

The observed difference between the LoS distance of the AS and the NDS was expected; however, the higher LoS performance in amputees is a notable finding. This finding can be explained through several mechanisms. One possible explanation is that the assessment was conducted with participants in their self-selected, comfortable foot position, allowing amputees to achieve a wider base of support. Previous studies have reported that amputees tend to increase their base of support to enhance stability [41–44]. The fact that the center of gravity (CoG) of the support surface is an effective factor in displacement, and that in our study the static postural sway values of the TA group were higher than those of the control group, and the tendency to increase support surfaces may explain the increase in LoS on the amputated side. Additionally, greater weight bearing on the intact limb and increased CoP displacement toward this side during both static standing and LoS assessments are well documented compensatory strategies in amputees. This is because the intact side provides superior proprioceptive feedback and stability compared to the prosthesis, which is limited by mechanical and sensory constraints [45–47]. While amputees may utilize some support from the prosthesis when shifting toward the amputated side, this support is less effective than that provided by the intact limb. This can be explained by the reliance on the intact limb for stability, as well as the inability to obtain sufficient support from the prosthetic limb when moving toward the intact side, despite utilizing support from the intact limb during movements toward the prosthetic side [48–50]. Overall, these findings suggest that postural adjustments observed in amputees during LoS assessments are consistent with previously reported strategies to enhance stability, rather than indicating novel compensatory mechanisms.

In static stance, the lateral postural sway range was greater in the TA than in the CG in both CSEO and CSEC conditions. Damage to or reduced feedback from any sensory system due to disease or trauma can negatively affect balance [51]. The loss of sensory receptors below the knee following transtibial amputation likely contributes to reduced somatosensory feedback, which can affect balance. In addition, transtibial amputation may limit the individual’s ability to sense the ground beneath them and maintain balance [52]. For these reasons, somatosensory feedback reduction and sensory loss due to transtibial amputation may be the primary cause of static balance impairment in individuals with TA, particularly on compliant surfaces. Previous studies have demonstrated that individuals with transtibial amputation exhibit poorer static and dynamic balance control compared to healthy individuals [5,20]. Hermodsson et al. [53] compared postural sway in static stance under eyes open and eyes closed conditions between individuals with TA and CG. They found that the TA had significantly greater lateral postural sway than the CG group under eyes closed condition. Similarly, Isakov et al. [2] found that total postural sway during static standing was greater in the TA compared to the CG, particularly under eyes closed conditions. Fernie et al. [54] also observed increased postural sway under eyes closed conditions in lower limb amputees, attributing this to a greater reliance on visual input as an adaptive response to the loss of proprioception following amputation [55]. It has been shown that amputees may have difficulty using somatosensory information, and that this condition increases postural sway, particularly on perturbed surfaces where maintaining balance is more challenging [12]. In addition, studies indicate that amputees adopt distinct movement strategies to maintain postural balance, demonstrating greater reliance on lateral movement strategies [46,56]. These findings align with the results of the present study, which showed that the TA exhibited greater lateral sway than the CG under compliant surface conditions, both with eyes open and closed.

During the LoS assessment, the greater EO activation on the IS in the TA compared to the DS in the CG, as well as the greater EO activation on the IS compared to the AS in the TA, may suggest that the EO muscles on the IS play a compensatory role in addressing amputation-related deficiencies during shifts in the CoG. These findings may imply that the TA utilizes their EO muscles on the intact side more effectively, enabling controlled trunk shifts toward the amputated side and greater CoP displacement. In this study, it is thought that the higher EO muscle activation in TA on the intact side than on the amputated side during LoS measurements may be due to the greater CoP displacement of the participants towards the amputee side during LoS measurements. Following amputation, individuals tend to shift more weight onto the intact side, leading to increased activation of trunk muscles, particularly EO muscles among the muscles examined in this study. Insufficient muscle function on the amputated side may be compensated by greater activation of muscles on the intact side. Consequently, these findings are consistent with previous reports indicating that individuals with lower limb amputation exhibit increased activation of trunk muscles on the intact side during balance tasks [57–59]. This adaptive mechanism may appear to be achieved through increased activation of the EO muscles, which play a key role in trunk rotation and lateral flexion [60]. Additionally, the stabilization role of the less-preferred limb during lateral perturbations highlights the importance of eccentric control of contralateral muscles in maintaining balance and stability [61]. Thus, the greater EO activation on the intact side in the TA may reflect a biomechanical adaptation that facilitates postural stability by compensating for deficits on the amputated side.

In the TA, the MF EMG amplitude on the amputated side was higher than on the intact side under CSEO and CSEC conditions, with a statistically significant difference observed under CSEO. Consistent with our findings, Sions et al. [62] reported that MF activation on the intact side was 9% lower in their study involving 14 transtibial amputees. The greater MF activation on the amputated side during static postural control may be suggest that individuals with transtibial amputation use more on the posterior deep trunk muscles of the affected side to maintain balance. In addition, an elevated EMG amplitude indicates an increased neuromuscular demand, implying that greater effort is required from the neuromuscular system to compensate for postural instability in individuals with TA [63]. Overall, these results may indicate a possible adaptation in trunk muscle activation patterns in individuals with TA during postural control, developed in response to increased neuromuscular demands to maintain balance.

In our study, LD activation on the IS in TA was higher than in CG across NSEC, NSEO, CSEC, and CSEO conditions, while LD activation on the AS was also higher than CG levels at CSEC. Supporting our findings, a previous study assessing trunk muscle activation in lower limb amputees reported increased erector spinae (ES) activity during forward flexion tasks compared to controls [62]. It has been suggested that compensatory increases in ES activity during trunk movements after lower extremity amputation are due to decreased passive structural support [64,65]. Similarly, biomechanical studies have shown higher ES activation on the intact side in individuals with unilateral lower limb amputation compared to controls [57,66]. These findings have been linked to excessive lateral bending toward the amputated side [66], increased rotational angular momentum [67], and reduced mechanical resistance of the trunk (trunk stiffness) on the amputated side [57]. Individuals with TA may be attempting to adapt trunk stiffening compensation to maintain balance by limiting perturbations of the body center of mass (CoM). This protective strategy also may serve to prevent secondary injuries to the spine [60,68]. Moreover, this strategy is consistent with other conservative strategies adopted by individuals with TA to prevent falls when loading the amputee limb during high-demand tasks such as walking on uneven surfaces [69], stairs [60,70], or inclined surfaces [71]. Accordingly, the reasons for the increased LD activation on the IS during postural sway assessments under all conditions in individuals with TA, as well as the greater LD activation on the AS during eyes-closed assessments on compliant surface, may be related to the need to increase trunk stiffness with adaptive strategies developed to ensure trunk stability and maintain balance. Finally, these findings may suggest a potential role for LD strengthening in future rehabilitation approaches aimed at improving postural control in individuals with TA, though further research is needed to confirm this.

Moreover, as mentioned above, in addition to the fact that the LD activation on the IS during all postural sway assessment conditions in individuals with TA was greater than that of the DS in the CG, its being greater than that of the AS during CSEO may be due to lower LD muscle strength on the IS. This finding is also supported by the information suggesting that, during gait, increased effort of the trunk muscles on the amputated side may be required to compensate for hip torque deficiencies on that side [62], and that this may result in muscle hypertrophy and improved muscle quality on the amputated side [72]. Because increased trunk muscle strength and hypertrophy on the amputated side, along with relatively lower strength of the LD muscle on the intact side, may necessitate greater muscle activation. With this, when considering only the CSEO condition, it was observed that LD activation was greater on the IS in individuals with TA, and that the LD activation on the IS in the TA group was also greater than that of the DS in the CG. Moreover, in contrast, it was determined that MF muscle activation on the AS in individuals with TA was significantly higher compared to the IS. These findings may suggest that the increase in activation of the LD muscle (one of the superficial spinal stabilizer muscles belonging to the erector spinae group) in individuals with TA may compensate for the decrease in MF activation (a deep spinal stabilizer) [62,64]. From a balance perspective, automatic contractions of the deep spinal stabilizers are necessary for postural control [62]. Therefore, the decreased MF activation on the intact side in individuals with TA may also help explain the findings of Hendershot et al. [73], who reported greater postural sway during sitting following lower limb amputation. In other populations, including healthy individuals and those with low back pain, interventions targeting posterior trunk muscle activation through motor control exercises such as side planks and rolling movements using a Swiss ball [74] have been effective in addressing reduced MF activation [75]. This suggests that exercises targeting deep trunk stabilizers may hold potential to influence MF activation, although further studies are needed to explore this in individuals with TA. Furthermore, the fact that LD activation on the intact side was higher in all postural sway assessment conditions in individuals with TA compared to the dominant side in the CG is thought to be associated with the involvement of the LD muscle – which provides posterior trunk stability on the intact side – in TA, and with an effort to achieve control through increased activation of this muscle, which may be insufficient for maintaining balance during postural control. This finding suggest that changes in trunk muscle activation patterns may occur in individuals with TA during postural control tasks. With training and familiarity, excessive trunk muscle demand may be reduced, potentially contributing to more efficient postural control strategies over time [76,77]. However, this remains to be investigated in future longitudinal studies. In conclusion, this study provides preliminary insights into postural control and compensatory strategies in individuals with transtibial amputations. Given its exploratory nature and the small sample size, the results should be interpreted with caution. As a pilot study, these findings will guide larger-scale future research that can more definitively assess the clinical implications.

### 4.1 Limitations

A significant limitation of this study is the relatively small sample size, inherent to research involving a specific population of unilateral transtibial amputees. This restricts the generalizability of the findings and prevents reliable conclusions regarding clinical relevance. It is important to note that, this study was designed and conducted as a pilot investigation therefore, the results should be interpreted as preliminary and exploratory. The purpose of this pilot work is to provide initial insights and inform the design of larger, adequately powered studies that can evaluate the observed patterns more robustly. Additionally, the findings of this study can only be generalized to male individuals who have undergone unilateral transtibial amputation due to traumatic causes and use a prosthetic leg. With this, setting the upper age limit at 45 to control for age-related and age-increasing physiological and neuromuscular variability and ensure sample homogeneity limits the study’s ability to represent a broader population. In the LoS assessment, the fact that EMG signals were not analyzed separately for each direction and all movement directions were reported collectively constitutes a limitation of the study. Another limitation of this study is that the potential effects of psychological or behavioral factors related to amputation were not directly measured.

### 4.2 Future directions

Future research should explore the link between trunk muscle activation and functional tasks, including daily and sport-specific activities, in transtibial amputees. This study highlights compensatory mechanisms and postural adaptations, emphasizing the need for targeted rehabilitation to improve balance and stability. These findings can help refine movement patterns and rehabilitation strategies. Additionally, investigating rehabilitation programs that enhance trunk muscle use on the affected side will be essential for addressing asymmetrical muscle imbalances in this population. Given the pilot nature of the present study, future investigations should build on these preliminary findings by recruiting larger, adequately powered, and more diverse samples, which will allow for robust statistical analyses and more clinically relevant conclusions. Future studies conducted with larger sample sizes, including different age groups and female participants, will ensure a broader representation of the population. Furthermore, future studies should include direct assessments of psychological and behavioral factors such as anxiety levels and balance confidence to gain a more comprehensive understanding of their effects on postural control and trunk muscle activation in individuals with transtibial amputations. Future research should comprehensively examine neuromuscular interaction through the integration of muscle structure (ultrasound measurements), motor unit analyses, peripheral nerve conduction, postural control modeling, and neurophysiological tests. In this way, it will be possible to interpret not only changes at the muscle level but also their integration with central and peripheral mechanisms. Finally, future research is recommended to evaluate additional kinetic and kinematic parameters in addition to the CoP. In this way, postural control mechanisms can be defined more comprehensively.

## 5. Conclusions

This study is one of the first to examine the relationship between trunk muscle activation and postural control following transtibial amputation. Asymmetric trunk muscle activations are observed during balance tasks in individuals with transtibial amputation. Impaired trunk muscle activity and balance performance, both under eyes open and eyes closed conditions, may represent modifiable parameters and warrant further investigation in rehabilitation contexts.

Our study emphasizes the critical role of trunk muscles in maintaining and improving balance in individuals who have undergone unilateral transtibial amputation. These preliminary findings may support the future inclusion of trunk muscle training as part of rehabilitation strategies, aimed at improving balance, preventing complications that may affect the trunk due to asymmetric movement patterns, enhancing daily living activities, and improving quality of life in this population. However, further studies with larger samples are needed to confirm these observations.

## Supporting information

S1 Data Raw data used for the statistical analyses presented in the manuscript. (SAV)

S2 Data Processed dataset used to generate the main figures and tables. (CSV)

S3 Checklist Completed reporting checklist according to the journal’s guidelines. (DOCX)
