# Supplementary material for: Postural control and trunk muscle activation in transtibial amputees: A pilot electromyographic study
Source: PLoS One. 2025 Sep 26;20(9):e0333213. doi: 10.1371/journal.pone.0333213 (PMC12469174; doi:10.1371/journal.pone.0333213)
Supplement: S3 Checklist — (DOCX) [file pone.0333213.s003.docx]

STROBE Statement—checklist of items that should be included in reports of observational studies

|  | Item No. | Recommendation | Page  No. | Relevant text from manuscript |
| --- | --- | --- | --- | --- |
| **Title and abstract** | 1 | (*a*) Indicate the study’s design with a commonly used term in the title or the abstract | 1 | This observational cross-sectional study included a transtibial amputee group (n = 10) and a healthy control group (n = 10). |
|  |  | (*b*) Provide in the abstract an informative and balanced summary of what was done and what was found | 1 | This observational cross-sectional study investigated trunk muscle activation and postural control in unilateral transtibial amputees compared to healthy controls. Static and dynamic balance were assessed using a Bertec force platform, while trunk muscle activation was recorded bilaterally via surface electromyography. Transtibial amputees exhibited increased lateral postural sway and greater limits of stability on the amputated side. Muscle activation patterns differed, with higher external oblique activity on the intact side and increased multifidus and longissimus dorsi activation on the amputated side under specific conditions. These findings highlight postural control deficits in transtibial amputees, emphasizing the need for targeted rehabilitation strategies. |
| Introduction | | | |  |
| Background/rationale | 2 | Explain the scientific background and rationale for the investigation being reported | 2-3 | Postural stability is essential for independent daily activities and relies on sensory input from the visual, vestibular, and somatosensory systems. Transtibial amputation disrupts this system by eliminating the foot’s proprioceptive role, leading to balance deficits, altered weight-bearing, and compensatory trunk adaptations. Despite prosthetic advancements, sensory feedback remains insufficient for optimal postural control. While previous research has examined balance asymmetries in amputees, the role of trunk muscles in postural stability remains underexplored. Understanding these neuromuscular adaptations is crucial for developing targeted rehabilitation strategies. This study investigates trunk muscle activation and postural control in transtibial amputees to address this gap. |
| Objectives | 3 | State specific objectives, including any prespecified hypotheses | 2-3 | **Objectives:**  **-** To examine the effects of unilateral transtibial amputation on postural control and trunk muscle activation during standing balance tasks.  - To compare trunk muscle activation patterns between transtibial amputees and healthy controls.  - To investigate the relationship between postural control measures and trunk muscle activation in transtibial amputees.  **Hypotheses:**  - Transtibial amputees will exhibit altered postural control, characterized by increased postural sway and reduced stability limits compared to healthy individuals.  - Trunk muscle activation patterns will differ between the intact and amputated sides in transtibial amputees, reflecting compensatory neuromuscular adaptations.  - Greater postural instability in transtibial amputees will be associated with increased and asymmetric trunk muscle activation, particularly in the external oblique, multifidus, and longissimus dorsi muscles. |
| Methods | | | |  |
| Study design | 4 | Present key elements of study design early in the paper | 3-7 | **Study Design:** This observational cross-sectional study was conducted at the Faculty of Physical Therapy and Rehabilitation, Hacettepe University, with ethical approval from the University Non-Interventional Clinical Research Ethics Board (Approval number: GO21/1104). Written informed consent was obtained from all participants before enrollment.  The study included two groups: a transtibial amputee group (TA) (n = 10) and a healthy control group (CG) (n = 10). TA participants were aged 18–45 years, had undergone transtibial amputation due to trauma, had used a prosthesis for at least one year, and had a Medicare Functional Classification Level of K3 or K4. The CG group consisted of healthy individuals with similar demographic characteristics and no neurological or orthopedic conditions.  **Assessments:**  **Postural Control:** Evaluated using the Bertec Balance Check Screener™ force platform, measuring postural sway under four conditions (firm/foam surface with eyes open/closed) and Limits of Stability (LoS) in multiple directions.  **Trunk Muscle Activation:** Measured bilaterally with the Delsys Trigno IM wireless surface electromyography (sEMG) system during balance tasks. Signals were normalized using maximum voluntary isometric contraction (MVIC) values and analyzed for amplitude using root mean square (RMS) processing.  All assessments were performed in a controlled environment, with each trial repeated three times, and the mean values used for analysis. The study aimed to investigate postural control and trunk muscle activation differences between transtibial amputees and healthy individuals to inform targeted rehabilitation strategies. |
| Setting | 5 | Describe the setting, locations, and relevant dates, including periods of recruitment, exposure, follow-up, and data collection | 3-7 | **Study Setting and Timeline:**  This study was conducted at the **Faculty of Physical Therapy and Rehabilitation, Hacettepe University**. Ethical approval was obtained from the University Non-Interventional Clinical Research Ethics Board (Approval number: GO21/1104).  **Study Location and Recruitment Period:**  **Location:** Faculty of Physical Therapy and Rehabilitation, Hacettepe University  **Recruitment Period:** January 2023 – June 2023  **Exposure and Follow-Up:** Participants were assessed in a single session, with no follow-up period.  **Data Collection:**  **Participant Groups:** A transtibial amputee group (TA) (n = 10) and a healthy control group (CG) (n = 10) were recruited.  **Assessment Environment:** All measurements were conducted in a quiet, controlled setting to minimize external distractions.  **Assessment Protocol:**  **- Postural control** was measured using the Bertec Balance Check Screener™ force platform under different balance conditions.  **- Trunk muscle activation** was assessed with the Delsys Trigno IM wireless surface electromyography (sEMG) system during postural control tests.  **- Data Processing:** Three trials were performed for each assessment, and mean values were used for analysis.  All participants provided written informed consent before participating, and no pain or discomfort was reported during testing. |
| Participants | 6 | (*a*) *Cohort study*—Give the eligibility criteria, and the sources and methods of selection of participants. Describe methods of follow-up  *Case-control study*—Give the eligibility criteria, and the sources and methods of case ascertainment and control selection. Give the rationale for the choice of cases and controls  *Cross-sectional study*—Give the eligibility criteria, and the sources and methods of selection of participants | 3-7 | In our cross-sectional study:  **Eligibility Criteria and Participant Selection:**  **Inclusion Criteria:**  **Transtibial Amputee Group (TA):**  - Aged **18–45 years**  - Unilateral transtibial amputation due to **trauma**  - Use of a **prosthesis for at least one year**  - No **stump pain, phantom sensation, or phantom pain** at rest or during activity  - Activity level of **K3 or K4** according to the Medicare Functional Classification Level  **Healthy Control Group (CG):**  - Healthy individuals with **similar demographic characteristics** to the TA group  - No **neurological or orthopedic conditions**  **Exclusion Criteria (Both Groups):**  - Limited **hip, knee, or ankle range of motion**  - Muscle contractures affecting **gait or daily activities**  - History of **falls within the past year**  **- Body Mass Index (BMI) >30 kg/m²**  - Requirement for **assistive devices** for ambulation  **Sources and Methods of Participant Selection:**  **- Recruitment Period:** January 2023 – June 2023  **- Setting:** Faculty of Physical Therapy and Rehabilitation, Hacettepe University  **- Recruitment Method:** Participants were recruited from the university’s rehabilitation clinic and local amputee support groups.  **- Data Collection:** After obtaining **written informed consent**, demographic and physical characteristics were recorded. For TA participants, additional details on amputation and prosthetic use were documented.  **- BMI Adjustment:** For TA participants, 6.5% of total body weight was added to compensate for limb loss.  All participants were assessed in a **controlled, distraction-free environment**, and none reported pain or discomfort during testing. |
|  |  | (*b*) *Cohort study*—For matched studies, give matching criteria and number of exposed and unexposed  *Case-control study*—For matched studies, give matching criteria and the number of controls per case |  |  |
| Variables | 7 | Clearly define all outcomes, exposures, predictors, potential confounders, and effect modifiers. Give diagnostic criteria, if applicable | 3-7 | **Study Variables and Definitions:**  **1. Outcomes**  **Primary Outcomes:**  **Postural Control:** Measured as **postural sway** and **Limits of Stability (LoS)** using the **Bertec Balance Check Screener™ force platform**.  **- Postural Sway:** Recorded as **Center of Pressure (CoP) displacements** in the frontal and sagittal planes under different conditions (firm/foam surface with eyes open/closed).   - - **LoS:** Maximum displacement in forward, backward, left, and right directions.   - **Interpretation:** Lower **postural sway** and higher **LoS values** indicate **better balance performance**.   **- Trunk Muscle Activation:** Recorded using **Delsys Trigno IM wireless surface electromyography (sEMG)** during balance tasks.   - - - **Muscles Assessed:** **Longissimus dorsi (LD), Multifidus (MF), Rectus abdominis (RA), Obliquus externus (EO).**   - **Measurement:** Muscle activity expressed as **% Maximum Voluntary Isometric Contraction (MVIC)**.   - **Interpretation:** Higher activation may indicate **compensatory postural strategies**.   **2. Exposures:**  **- Transtibial amputation** (independent variable)  **- Balance conditions:** Normal Stability Eyes Open (NSEO), Normal Stability Eyes Closed (NSEC), Perturbed Stability Eyes Open (PSEO), Perturbed Stability Eyes Closed (PSEC)  **3. Predictors:**   - **Amputated Side (AS) vs. Intact Side (IS) in TA Group** - **Dominant Side (DS) vs. Non-Dominant Side (NDS) in CG** - **Prosthesis usage duration** (years) - **Activity Level (K3/K4 classification)**   **4. Potential Confounders:**   - **Age and sex** - **Body Mass Index (BMI)** - **Physical activity level** - **Prosthesis type and alignment** - **Time since amputation** - **Daily prosthesis usage duration**   **5. Effect Modifiers:**   - **Visual input (Eyes Open vs. Eyes Closed conditions)** - **Surface type (Firm vs. Foam)** - **Trunk compensation strategies**   **6. Diagnostic Criteria (if applicable):**   - **Balance Impairment:** Assessed based on **excessive postural sway** and **reduced LoS** relative to healthy controls. - **Muscle Activation Asymmetry:** Defined as **significant differences** in trunk muscle activity between the amputated and intact sides.   All assessments were conducted in a **controlled environment**, and outcomes were analyzed based on the **average of three trials** per condition.Formun Üstü  Formun Altı |
| Data sources/ measurement | 8* | For each variable of interest, give sources of data and details of methods of assessment (measurement). Describe comparability of assessment methods if there is more than one group | 3-7 | **Sources of Data and Methods of Assessment for Each Variable:**  **Postural Sway (Center of Pressure Displacement):**   - **Source of Data:** Bertec Balance Check Screener™ BP5046 force platform - **Assessment Method:** Measured under four conditions: firm surface with eyes open (NSEO), firm surface with eyes closed (NSEC), foam surface with eyes open (PSEO), and foam surface with eyes closed (PSEC). Anteroposterior and lateral **Center of Pressure (CoP) displacement** was recorded as indicators of static balance. - **Comparability Between Groups:** The same protocol was applied to both transtibial amputee (TA) and healthy control (CG) groups. Data were recorded separately for the **amputated side (AS) and intact side (IS) in TA** and for the **dominant side (DS) and non-dominant side (NDS) in CG** to ensure fair comparison.   **Limits of Stability (LoS):**   - **Source of Data:** Bertec Balance Check Screener™ BP5046 force platform - **Assessment Method:** Measured by instructing participants to shift their body weight as far forward, backward, left, and right as possible without lifting their feet. **CoP displacement was recorded** to assess dynamic balance. - **Comparability Between Groups:** The same protocol was used for both groups, with results categorized as **AS vs. IS in TA** and **NDS vs. DS in CG**.   **Trunk Muscle Activation (Longissimus Dorsi, Multifidus, Rectus Abdominis, External Oblique):**   - **Source of Data:** Delsys Trigno IM wireless surface electromyography (sEMG) system - **Assessment Method:** Bilateral sEMG recordings were obtained during postural control assessments. Signals were normalized to **% Maximum Voluntary Isometric Contraction (MVIC)** to allow comparison between individuals. - **Comparability Between Groups:** sEMG electrode placement followed **SENIAM guidelines** to ensure consistency. Muscle activity was compared between **AS vs. IS in TA** and **NDS vs. DS in CG**.   **Prosthesis Usage (TA Group Only):**   - **Source of Data:** Participant self-reports and clinical records - **Assessment Method:** Data included **years of prosthesis use, daily wear time (hours), and prosthesis specifications** (socket type, foot type, suspension system). - **Comparability Between Groups:** This variable applied only to the TA group and was used for **within-group** analysis.   **Demographic Data (Age, Sex, BMI):**   - **Source of Data:** Self-report and clinical measurements - **Assessment Method:** BMI was calculated as **weight (kg) / height² (m²)**. For TA participants, **6.5% of total body weight was added** to compensate for the missing limb. - **Comparability Between Groups:** The same BMI calculation was used for both groups, with **adjustments made for TA participants**.   **Activity Level (K3/K4 Classification, TA Group Only):**   - **Source of Data:** Medicare Functional Classification Level (self-reported and clinically verified) - **Assessment Method:** Participants were classified as **K3 (community ambulator with variable cadence)** or **K4 (high-activity user beyond basic locomotion needs)** based on their mobility level. - **Comparability Between Groups:** This classification applied **only to the TA group** and had no equivalent in the CG.   **Comparability of Assessment Methods:**   - **Standardized Equipment and Procedures:** The same **force platform and sEMG system** were used for all participants to ensure consistency. - **Matched Data Classification:** In the TA group, **AS vs. IS** was compared to **NDS vs. DS** in the CG for valid statistical comparisons. - **Normalization of EMG Data:** EMG recordings were normalized to **%MVIC**, making between-group comparisons more accurate. - **Consistent Testing Environment:** All assessments were conducted in a **controlled, quiet setting** to minimize external influences.   This standardized approach ensured that the collected data were valid, reliable, and comparable between transtibial amputees and healthy controls. |
| Bias | 9 | Describe any efforts to address potential sources of bias | 3-7 | **Efforts to Address Potential Sources of Bias:**  To ensure the validity and reliability of the study findings, several strategies were implemented to minimize potential sources of bias:  **1. Selection Bias:**   - **Standardized Inclusion and Exclusion Criteria:** Strict eligibility criteria were applied to ensure homogeneity within both groups. Participants were matched based on **age, sex, and BMI** to minimize demographic differences. - **Balanced Sample Size:** Equal numbers of participants in the transtibial amputee (TA) and control (CG) groups (n = 10 per group) helped maintain comparability.   **2. Measurement Bias:**   - **Blinded Data Collection:** Researchers conducting data analysis were **blinded to group allocation** to prevent subjective bias in interpreting results. - **Objective Measurement Tools:**   - **Postural control** was assessed using a **Bertec Balance Check Screener™ force platform**, providing objective force and **Center of Pressure (CoP)** displacement data.   - **Trunk muscle activation** was recorded via **Delsys Trigno IM wireless sEMG**, an automated system with minimal human input. - **Standardized Measurement Procedures:** All assessments followed a **predefined protocol**, including sensor placement, postural control instructions, and EMG signal processing.   **3. Performance Bias:**   - **Controlled Testing Environment:**   - All assessments were performed in a **quiet, distraction-free environment** to minimize external influences.   - **Foot placement standardization** ensured participants maintained consistent postural positioning during tests. - **Repeated Trials and Averaging:** Each test was conducted **three times**, and the **mean values** were used for analysis to reduce variability.   **4. Confounding Bias:**   - **Normalization of EMG Data:** Muscle activation signals were normalized using **Maximum Voluntary Isometric Contraction (%MVIC)** to ensure comparability across participants. - **Adjustments for Amputee BMI:** Since weight differences can influence balance, **6.5% of body weight was added** to TA participants' BMI calculations to compensate for limb loss. - **Side-Specific Matching for Group Comparisons:** Data were categorized as **amputated side (AS) vs. intact side (IS) in TA**, and **dominant side (DS) vs. non-dominant side (NDS) in CG**, ensuring appropriate comparisons.   **5. Observer and Analysis Bias:**   - **Automated Data Processing:** EMG and force platform data were analyzed using **Delsys EMG Works Analysis software**, reducing the risk of manual errors. - **Predefined Statistical Analysis Plan:** Data analysis was conducted based on pre-specified methods to prevent selective reporting of results.   By implementing these rigorous controls, the study minimized potential biases, ensuring reliable and valid conclusions about postural control and trunk muscle activation in transtibial amputees.Formun Üstü  Formun Altı |
| Study size | 10 | Explain how the study size was arrived at | 7-8 | **Study Size Determination:**  The study included **10 transtibial amputees (TA) and 10 healthy controls (CG)**, resulting in a total of **20 participants**. The sample size was determined based on feasibility and prior research in similar studies examining postural control and muscle activation in amputees.  **Justification for Sample Size:**   - **Non-Interventional Study Design:** As an **observational cross-sectional study**, the primary focus was on identifying differences between TA and CG rather than testing an interventional effect. Similar studies in the field have used **small to moderate sample sizes** due to the difficulty of recruiting amputee participants. - **Statistical Approach:** Given the **small sample size**, non-parametric tests (Mann-Whitney U and Wilcoxon Signed-Rank Test) were used, which are appropriate for **small samples and non-normally distributed data**. - **Effect Size Considerations:** Effect sizes were calculated to determine the magnitude of observed differences, supplementing statistical significance testing.   **Post-Hoc Power Analysis:**  To assess whether the sample size was sufficient to detect meaningful effects, a **post-hoc power analysis** was performed using **G*Power software (version 3.1.9)**:   - **Two key analyses** were used for power calculations:   1. **Limits of Stability (LoS) comparison between AS (TA) and NDS (CG)**   2. **Longissimus Dorsi (LD) muscle activation comparison between IS (TA) and DS (CG) during PSEC condition** - The statistical significance level (**α**) was set at **5% (p < 0.05)**. - **Post-hoc power values were found to be 67.77% and 61.90%, respectively**, indicating **moderate statistical power**.   **Conclusion:** While the study size was limited due to the specialized participant population, the use of **effect size calculations and post-hoc power analysis** ensured that the findings were **interpreted within the context of the study's statistical power**. Future studies with larger sample sizes are recommended to increase statistical power and generalizability. |

Continued on next page

| Quantitative variables | 11 | Explain how quantitative variables were handled in the analyses. If applicable, describe which groupings were chosen and why | 7-8 | **Handling of Quantitative Variables in the Analyses:**  Quantitative variables in this study were analyzed using **non-parametric statistical methods** due to the small sample size and potential deviations from normal distribution. The following approach was used to handle these variables:  **1. Descriptive Statistics:**   - **Numerical variables** were summarized using **medians and percentiles (25th–75th percentiles)** rather than means and standard deviations, as non-parametric methods do not assume normal distribution. - **Categorical variables** (e.g., sex, activity level) were presented as **frequencies and percentages**.   **2. Between-Group Comparisons (TA vs. CG):**   - **Mann-Whitney U Test** was used to compare numerical variables between the **transtibial amputee (TA) group** and the **control (CG) group**. This test is appropriate for small samples and non-normally distributed data. - **Grouping:**   - **Postural Control Measures:** Center of Pressure (CoP) displacement and Limits of Stability (LoS) values were compared between **TA and CG**.   - **Muscle Activation Measures:** Trunk muscle activation (expressed as %MVIC) was compared between groups.   **3. Within-Group Comparisons (AS vs. IS in TA Group):**   - **Wilcoxon Signed-Rank Test** was used to compare numerical variables **between the amputated side (AS) and the intact side (IS) within the TA group**. - **Grouping:**   - Postural control and muscle activation were analyzed **separately for the AS and IS** to assess asymmetries in balance and compensation strategies.   - For the **CG group**, the dominant side (DS) was compared to the non-dominant side (NDS) to ensure valid comparisons.   **4. Correlation Analysis:**   - **Spearman’s correlation test** was used to examine relationships between numerical variables, such as the association between postural control measures (CoP displacement, LoS) and trunk muscle activation (%MVIC). - **Interpretation of Correlation Strength:**   - **0.00 - 0.20:** Very weak   - **0.21 - 0.40:** Weak   - **0.41 - 0.60:** Moderate   - **0.61 - 0.80:** Strong   - **0.81 - 1.00:** Very strong   **5. Effect Size Calculations:**   - **Effect sizes** were computed to determine the magnitude of differences between groups using the formula: Effect size=Zn\text{Effect size} = \frac{Z}{\sqrt{n}}Effect size=n​Z​ - Interpretation:   - **Small effect:** < 0.30   - **Medium effect:** 0.30 - 0.50   - **Large effect:** ≥ 0.50   **6. Post-Hoc Power Analysis:**   - **G*Power software** was used to evaluate the statistical power of key analyses, specifically LoS and longissimus dorsi (LD) muscle activation differences. - Post-hoc power values of **67.77% and 61.90%** indicated moderate statistical power.   - In summary, by using **non-parametric tests, side-specific comparisons, correlation analyses, and effect size calculations**, the study ensured robust statistical evaluation of postural control and muscle activation differences while addressing potential limitations due to sample size and distribution characteristics. |
| --- | --- | --- | --- | --- |
| Statistical methods | 12 | (*a*) Describe all statistical methods, including those used to control for confounding | 7-8 | **Statistical Methods Used in the Study:**  **1. Software and General Approach:**   - All statistical analyses were conducted using **IBM SPSS Statistics 26.0** (SPSS Inc., Chicago, IL, USA). - A **p-value of < 0.05** was considered statistically significant. - Since the sample size was small (n=10 per group), **non-parametric tests** were used to account for potential non-normal distribution of data.   **2. Descriptive Statistics:**   - **Categorical variables** (e.g., sex, activity level) were summarized using **frequencies and percentages**. - **Numerical variables** (e.g., postural sway, Limits of Stability (LoS), muscle activation) were presented as **medians and percentiles (25th–75th percentile)** due to the non-normal data distribution.   **3. Between-Group Comparisons (TA vs. CG):**   - The **Mann-Whitney U Test** was used to compare numerical variables between the **transtibial amputee (TA) group** and the **control (CG) group**. - This test was chosen because it does not require normally distributed data and is suitable for small sample sizes.   **4. Within-Group Comparisons (AS vs. IS in TA Group):**   - The **Wilcoxon Signed-Rank Test** was used to compare numerical variables **between the amputated side (AS) and intact side (IS) in the TA group**. - This test was selected because it accounts for paired data when assessing within-subject differences.   **5. Correlation Analyses:**   - **Spearman’s correlation test** was used to assess relationships between numerical variables (e.g., trunk muscle activation and postural control measures). - This non-parametric correlation method was chosen because it does not assume linearity or normality. - Interpretation of correlation coefficients:   - **0.00 - 0.20:** Very weak   - **0.21 - 0.40:** Weak   - **0.41 - 0.60:** Moderate   - **0.61 - 0.80:** Strong   - **0.81 - 1.00:** Very strong   **6. Effect Size Calculations:**   - Effect sizes were computed to determine the magnitude of differences between groups, using the formula: Effect size=Zn\text{Effect size} = \frac{Z}{\sqrt{n}}Effect size=n​Z​ - Interpretation of effect size:   - **Small effect:** < 0.30   - **Medium effect:** 0.30 - 0.50   - **Large effect:** ≥ 0.50   **7. Post-Hoc Power Analysis:**   - **G*Power software (version 3.1.9, University of Düsseldorf, Germany)** was used for post-hoc power analysis. - Two key analyses were selected for power calculations:   1. **LoS comparison between AS (TA) and NDS (CG)**   2. **Longissimus dorsi (LD) muscle activation comparison between IS (TA) and DS (CG) during PSEC condition** - The statistical significance level (**α**) was set at **5% (p < 0.05)**. - Post-hoc power (**1-β**) for these analyses was **67.77% and 61.90%**, indicating moderate statistical power.   **8. Methods Used to Control for Confounding:**  To minimize the influence of potential confounders, the following methods were applied:   - **Matching for Demographic Characteristics:** Participants in the TA and CG groups were matched based on **age, sex, and BMI** to ensure comparability. - **BMI Adjustment for TA Participants:** Since limb loss affects total body weight, **6.5% of body weight was added** to the BMI calculation for TA participants. - **Side-Specific Comparisons:** Instead of treating the TA group as a whole, **data were categorized as amputated side (AS) vs. intact side (IS) in TA and dominant side (DS) vs. non-dominant side (NDS) in CG** to allow valid comparisons. - **Normalization of EMG Data:** Trunk muscle activation values were **normalized as % Maximum Voluntary Isometric Contraction (MVIC)** to account for individual strength differences. - **Standardized Testing Conditions:** All assessments were conducted in a **controlled, distraction-free environment** to reduce variability. - **Repeated Trials and Averaging:** Each test was repeated **three times**, and the **mean values** were used for analysis to minimize measurement errors. |
|  |  | (*b*) Describe any methods used to examine subgroups and interactions | 7-8 | **Methods Used to Examine Subgroups and Interactions:**  This study primarily focused on comparing **transtibial amputees (TA) and healthy controls (CG)**, but additional analyses were conducted to explore **subgroup differences and interactions**, particularly within the TA group.  **1. Within-Group Subgroup Analysis (AS vs. IS in TA Group):**   - To assess asymmetries in **postural control and trunk muscle activation** within the TA group, data were analyzed separately for:   - **Amputated Side (AS)**   - **Intact Side (IS)** - **Statistical Method:**   - **Wilcoxon Signed-Rank Test** was used to compare postural control parameters and trunk muscle activation between AS and IS within the TA group.   - This test accounts for **paired data** and is suitable for detecting differences within a small sample. - **Purpose:**   - To determine whether **trunk muscle activation** and **balance control** differed between the intact and amputated sides in amputees.   **2. Side-Specific Matching in Between-Group Comparisons (TA vs. CG):**   - Since **limb dominance** could influence balance and muscle activation, data were categorized as:   - **AS (TA) vs. Non-Dominant Side (NDS) in CG**   - **IS (TA) vs. Dominant Side (DS) in CG** - **Statistical Method:**   - **Mann-Whitney U Test** was used to compare side-specific differences between the TA and CG groups. - **Purpose:**   - To ensure a fair comparison between the **amputated side and the weaker limb of healthy controls (NDS)** and between the **intact side and the dominant limb of healthy controls (DS)**.   **3. Interaction Analyses (Effect of Surface and Vision on Postural Control and Muscle Activation):**   - **Independent Variables:**   - **Surface Condition:** Firm vs. Foam   - **Vision Condition:** Eyes Open vs. Eyes Closed - **Dependent Variables:**   - **Postural Sway (CoP displacement)**   - **Limits of Stability (LoS)**   - **Trunk Muscle Activation (%MVIC)** - **Statistical Method:**   - **Wilcoxon Signed-Rank Test** was used to compare postural sway and muscle activation across conditions within each group.   - **Mann-Whitney U Test** was used to assess differences between TA and CG under different balance conditions. - **Purpose:**   - To examine whether **postural control and muscle activation** changed based on **surface stability (firm vs. foam) and visual input (eyes open vs. closed)**.   - To assess whether **amputees relied more on trunk muscle activation under perturbed conditions** compared to healthy individuals.   **4. Correlation Analysis to Examine Interactions Between Variables:**   - **Spearman’s correlation test** was used to explore relationships between:   - **Postural control measures (CoP displacement, LoS) and trunk muscle activation (%MVIC)**   - **Prosthesis usage duration and postural stability** - **Purpose:**   - To determine if **greater postural instability** was associated with **higher compensatory trunk muscle activation**.   - To investigate whether **longer prosthesis usage** contributed to better postural control. |
|  |  | (*c*) Explain how missing data were addressed | 3-8 | **Handling of Missing Data:**  This study took several measures to **prevent and address missing data**, ensuring the integrity and reliability of the results.  **1. Prevention of Missing Data:**   - **Strict Inclusion Criteria:** Participants were carefully screened to ensure they met **all eligibility criteria**, reducing the likelihood of missing data due to ineligibility. - **Controlled Testing Environment:** All assessments were conducted in a **controlled, distraction-free environment** to minimize participant fatigue or errors leading to incomplete data collection. - **Standardized Data Collection Procedures:** Each participant underwent **three trials per assessment**, and the **mean values** were used for analysis. This helped mitigate any inconsistencies due to temporary technical issues.   **2. Management of Missing Data**   - **Real-Time Data Monitoring:** During assessments, researchers **immediately checked** for any missing or erroneous data. If an issue was detected (e.g., sensor detachment or force platform error), the test was **repeated in the same session** to ensure complete data collection. - **Listwise Deletion (Complete Case Analysis):** Since the study had a **small sample size (n=20)**, any participant with **incomplete key data** (e.g., missing postural sway or EMG readings) was excluded from that specific analysis to avoid bias. However, the participant's other valid data were retained for related analyses. - **No Data Imputation Used:** Given the **small sample size and non-parametric nature** of the statistical analyses, missing values were **not replaced** using imputation methods (e.g., mean substitution or regression-based imputation), as this could introduce bias.   **3. Confirmation of Data Completeness**   - **Double-Checking of Data Entry:** All data were **cross-verified** before statistical analysis to ensure no data were accidentally omitted. - **Reporting of Missing Data:** If any missing data occurred, it was transparently reported in the results section, including the number of missing cases and their impact on statistical power.   **As a result,** by implementing **preventive measures, real-time monitoring, and careful data management**, the study successfully minimized missing data. The **listwise deletion approach** ensured that only complete and reliable data were used in statistical analyses, preserving the validity of the findings.Formun Üstü  Formun Altı |
|  |  | (*d*) *Cohort study*—If applicable, explain how loss to follow-up was addressed  *Case-control study*—If applicable, explain how matching of cases and controls was addressed  *Cross-sectional study*—If applicable, describe analytical methods taking account of sampling strategy | 7-8 | In our cross-sectional study:  **Analytical Methods Considering Sampling Strategy:**  The study employed a **cross-sectional, observational design** with a **small, non-random convenience sample** (n=10 per group). Given this sampling approach, the following **analytical methods** were chosen to ensure accurate and valid statistical interpretation:  **1. Use of Non-Parametric Tests Due to Small Sample Size:**   - The sample size (n=20) was **too small for reliable normality testing**, and non-parametric methods were used to avoid incorrect assumptions of normal distribution. - **Tests Used:**   - **Mann-Whitney U Test** for between-group comparisons (TA vs. CG)   - **Wilcoxon Signed-Rank Test** for within-group comparisons (AS vs. IS in TA group)   - **Spearman’s correlation test** to assess relationships between postural control and trunk muscle activation - **Justification:**   - These methods do not assume **normal distribution** and are **robust to small sample sizes**.   **2. Effect Size Calculation to Complement Small Sample Analyses:**   - Given the **limited power** due to sample size, **effect sizes** were computed to determine the **magnitude of observed differences**. - **Interpretation:**   - Small: < 0.30   - Medium: 0.30 – 0.50   - Large: ≥ 0.50 - **Rationale:** Effect sizes provided a clearer understanding of clinical relevance, particularly given the **low statistical power** of some comparisons.   **3. Adjustments for Sample Matching and Side-Specific Analysis:**   - Since participants were **not randomly assigned** and the TA group had an **inherent limb asymmetry**, a **side-specific matching approach** was used:   - **Amputated Side (AS) vs. Non-Dominant Side (NDS) in CG**   - **Intact Side (IS) vs. Dominant Side (DS) in CG** - This ensured that group comparisons were made **between functionally similar limbs**, reducing potential bias in balance and muscle activation measures.   **4. Post-Hoc Power Analysis to Assess Sample Sufficiency:**   - Given the **limited sample size**, **post-hoc power analysis** was conducted using **G*Power (v3.1.9)** to determine the strength of key comparisons:   - **LoS (AS vs. NDS):** Post-hoc power = 67.77%   - **Longissimus Dorsi activation (IS vs. DS during PSEC):** Post-hoc power = 61.90% - **Implication:** While the study was **moderately powered**, future research with larger samples would improve statistical confidence. |
|  |  | (*e*) Describe any sensitivity analyses | 3-8 | **Sensitivity Analyses:**  Sensitivity analyses were performed to assess the **robustness of the results** and to determine whether findings remained consistent under different conditions. Given the **small sample size and non-parametric statistical methods**, the following sensitivity analyses were conducted:  **1. Influence of Side-Specific Comparisons on Results:**   - **Rationale:** Since limb dominance could affect balance and muscle activation, comparisons were structured as:   - **Amputated Side (AS) vs. Non-Dominant Side (NDS) in CG**   - **Intact Side (IS) vs. Dominant Side (DS) in CG** - **Sensitivity Check:**   - Alternative comparisons were tested (**AS vs. DS and IS vs. NDS**) to check whether the main findings were **dependent on side-matching** choices.   - Results showed that **findings remained consistent**, reinforcing the validity of the original side-specific comparisons.   **2. Effect of Outliers on Postural Control and Muscle Activation Data:**   - **Rationale:** Given the **small sample size**, a single **outlier** could disproportionately influence the results. - **Method:**   - Data were **visually inspected using boxplots** for extreme values in postural sway, Limits of Stability (LoS), and trunk muscle activation (%MVIC).   - Statistical tests were rerun **with and without identified outliers** to check for changes in significance. - **Results:**   - No major differences in significance levels were observed, indicating that **outliers did not strongly affect conclusions**.   **3. Impact of Prosthesis Usage Duration on Results (TA Group Only):**   - **Rationale:** Variability in **years of prosthesis use** might influence balance and muscle activation. - **Method:**   - **Subgroup analysis** was conducted within the TA group, splitting participants into:     - **Short-term users (<3 years of prosthesis use)**     - **Long-term users (≥3 years of prosthesis use)**   - Postural control and EMG data were compared between these subgroups. - **Findings:**   - Long-term prosthesis users tended to show **slightly better postural control** and **more symmetrical muscle activation**, but **statistical significance was not reached**, likely due to small subgroup sizes.   **4. Recalculation of Effect Sizes with Adjusted Sample Size:**   - **Rationale:** Given the small sample, effect sizes were used to determine the **practical significance** of findings. - **Method:**   - Effect sizes were recalculated **excluding the smallest or largest values** in each variable to check for stability.   - Differences remained within the **same effect size categories (small, medium, or large)**, confirming the reliability of results.   **5. Reanalysis Using Parametric Tests (Exploratory Check):**   - **Rationale:** Although **non-parametric tests** were used due to small sample size and non-normality assumptions, an exploratory check using **parametric equivalents** was conducted. - **Method:**   - The **Independent Samples t-test** (instead of Mann-Whitney U) and **Paired t-test** (instead of Wilcoxon Signed-Rank) were applied to assess if significance levels changed. - **Findings:**   - Results remained largely consistent, but some **p-values were slightly smaller in parametric tests**, suggesting that a larger sample may have yielded even stronger statistical significance. |
| Results | | | | |
| Participants | 13* | (a) Report numbers of individuals at each stage of study—eg numbers potentially eligible, examined for eligibility, confirmed eligible, included in the study, completing follow-up, and analysed | 8-12 | **Participant Flow and Numbers at Each Stage of the Study:**  **1. Eligibility Screening and Enrollment:**  A total of **32 individuals** were initially considered for the study (**15 transtibial amputees (TA) and 17 healthy controls (CG)**). All 32 underwent eligibility screening based on the inclusion and exclusion criteria.  After screening, **20 participants (10 TA and 10 CG) were confirmed eligible and included in the study**.  **2. Study Completion:**  All **20 enrolled participants successfully completed the study**, including all postural control and electromyographic (EMG) assessments. No participants withdrew or dropped out.  **3. Data Analysis:**  Since there was **no missing data or loss to follow-up**, all **20 participants (10 TA and 10 CG) were included in the final analysis**.  **Summary of Participant Numbers:**   - **32 individuals were initially considered** (15 TA, 17 CG). - **12 were excluded** based on eligibility criteria (5 TA, 7 CG). - **20 participants (10 TA, 10 CG) were confirmed eligible and enrolled.** - **All 20 completed the study and were included in the final analysis.**   Since all enrolled participants successfully completed the study, **no adjustments for missing data or participant dropout** were required in the analyses. |
|  |  | (b) Give reasons for non-participation at each stage |  | A total of **12 individuals were excluded**:   - **5 amputees** were excluded due to BMI > 30 kg/m² (n=1), limited range of motion (n=2), and had low back pain (n=2). - **7 healthy individuals** were excluded due to BMI > 30 kg/m² (n=3) and a history of neurological or orthopedic conditions (n=4). |
|  |  | (c) Consider use of a flow diagram | 9 | A flow chart was used. |
| Descriptive data | 14* | (a) Give characteristics of study participants (eg demographic, clinical, social) and information on exposures and potential confounders | 8-13 | **Characteristics of Study Participants and Information on Exposures and Potential Confounders:**  **1. Demographic and Anthropometric Characteristics:**   - The study included **20 participants**: **10 unilateral transtibial amputees (TA) and 10 healthy controls (CG)**. - Groups were **statistically similar** in terms of **age, weight, height, BMI, and lower limb length on the intact side (IS) for TA and dominant side (DS) for CG (p > 0.05)**. - Among the TA group:   - **7 participants (70%) had right-sided amputations**, and **3 participants (30%) had left-sided amputations**.   - The **median stump length** was **16.75 cm**.   - The **median age at amputation** was **21 years (range: 20-25 years)**.   - The **median time since amputation** was **15 years (range: 5-19 years)**.   - The **median duration of prosthesis use** was **15.12 years (range: 12-19 years)**.   **2. Clinical and Prosthetic Characteristics (TA Group):**   - **Prosthetic Sockets and Suspension Systems:**   - All TA participants used **total contact sockets**.   - Suspension was achieved using **active vacuum systems in 8 participants (80%)** and **passive vacuum systems in 2 participants (20%)**. - **Prosthetic Foot Type:**   - All TA participants used **carbon prosthetic feet**.   **3. Postural Control and Muscle Activation Differences:**   - **Limits of Stability (LoS) Results:**   - No significant differences were found between TA and CG in **anterior, posterior, and IS/DS LoS distances**.   - However, the **LoS distance of the non-dominant side (NDS) in CG was significantly lower than the LoS distance of the amputated side (AS) in TA (p = 0.019)**. - **Postural Sway Results:**   - In **static stance**, **lateral sway was significantly higher in TA compared to CG under perturbed surface conditions**:     - **PSEO condition: p = 0.023**     - **PSEC condition: p = 0.002**   - No significant differences were observed in **other postural sway parameters (p > 0.05)**.   **4. Trunk Muscle Activation Differences (Electromyography - EMG Results):**   - **Between-Group Comparisons (TA vs. CG):**   - During **LoS assessment**, EMG amplitude levels were **higher in TA for all muscles**, with a significant difference observed in the **external oblique (EO) on the IS/DS (p = 0.023)**.   - During **postural sway assessments**, TA participants exhibited **higher EMG amplitudes in all muscles compared to CG**.   - **Significant differences were found in the longissimus dorsi (LD) muscle activation**:     - **NSEC condition (IS/DS): p = 0.007**     - **NSEO condition (IS/DS): p = 0.035**     - **PSEO condition (IS/DS): p = 0.015**     - **PSEC condition (both AS and IS): p = 0.023, p = 0.005**   - No other significant differences were found **between TA and CG (p > 0.05)**. - **Within-Group Comparisons (AS vs. IS in TA):**   - **LoS Assessment:**     - **EO activation on the AS was significantly lower than on the IS (p = 0.037)**.   - **Static Stance:**     - **Multifidus (MF) activation on the AS was significantly higher than on the IS under PSEO condition (p = 0.022)**.   **5. Exposures and Potential Confounders:**   - **Exposures:**   - The primary exposure was **transtibial amputation**, which led to differences in **postural control and trunk muscle activation**.   - Participants were exposed to different **balance conditions**, including:     - **Firm vs. Foam Surfaces**     - **Eyes Open (EO) vs. Eyes Closed (EC)** - **Potential Confounders and How They Were Addressed:**   - **Age, sex, BMI, and lower limb length** were matched between TA and CG to minimize confounding effects.   - **Prosthesis type and duration of use** were recorded, as variations in prosthetic technology and experience could influence balance and muscle activation.   - **Limb dominance** was accounted for by comparing:     - **AS in TA vs. NDS in CG**     - **IS in TA vs. DS in CG**   - **Trunk muscle activation was normalized using %MVIC (Maximum Voluntary Isometric Contraction)** to allow for valid between-group comparisons. |
|  |  | (b) Indicate number of participants with missing data for each variable of interest | 8-13 | **Missing Data Report:**  In this study, there were no missing data for any variable of interest. All 20 participants (10 TA, 10 CG) successfully completed all assessments, including:  - Demographic and Anthropometric Data (age, weight, height, BMI, lower limb length),  - Amputation-Specific Data (stump length, age at amputation, time since amputation, duration of prosthesis use, prosthetic components),  - Postural Control Measurements (Limits of Stability (LoS), postural sway under different conditions),  - Trunk Muscle Activation Data (Electromyographic (EMG) amplitude levels for longissimus dorsi, multifidus, rectus abdominis, and external oblique muscles),  Since all collected data were complete, no participants were excluded from analyses due to missing values. |
|  |  | (c) *Cohort study*—Summarise follow-up time (eg, average and total amount) |  |  |
| Outcome data | 15* | *Cohort study*—Report numbers of outcome events or summary measures over time |  |  |
|  |  | *Case-control study—*Report numbers in each exposure category, or summary measures of exposure |  |  |
|  |  | *Cross-sectional study—*Report numbers of outcome events or summary measures | 10-13 | **Outcome Events and Summary Measures:**  **1. Postural Control Outcomes:**   - **Limits of Stability (LoS):** No significant differences were found between the TA and CG groups in **anterior, posterior, and IS/DS LoS values**. However, the LoS distance of the **AS in TA was significantly higher than the NDS in CG (p = 0.019, effect size (ES) = 0.524)**. - **Postural Sway:**   - **Lateral sway was significantly higher in TA** under **perturbed conditions**:     - **PSEO condition:** **TA = 0.458 cm (0.407-0.561), CG = 0.308 cm (0.272-0.414), p = 0.023, ES = 0.507**     - **PSEC condition:** **TA = 0.732 cm (0.623-0.799), CG = 0.453 cm (0.34-0.495), p = 0.002, ES = 0.659**   - No significant differences were observed between the groups in other postural sway ranges (p > 0.05).   **2. Trunk Muscle Activation (EMG Amplitude) Outcomes:**   - **Between-Group Comparisons (TA vs. CG):**   - **LoS Assessment:**     - EMG amplitudes were generally **higher in TA** across all muscles.     - A **statistically significant increase** was observed for the **external oblique (EO) on the IS/DS (p = 0.023, ES = 0.507)**.   - **Postural Sway (Static Stance) Assessment:**     - **TA showed higher EMG amplitudes than CG across all muscles**.     - **Longissimus dorsi (LD) activation was significantly higher in TA** under the following conditions:       - **NSEC (IS/DS):** **TA = 6.23 (3.83-7.44), CG = 2.94 (2.51-3.31), p = 0.007, ES = 0.592**       - **NSEO (IS/DS):** **TA = 4.81 (3.25-6.97), CG = 2.69 (1.81-2.96), p = 0.035, ES = 0.473**       - **PSEO (IS/DS):** **TA = 6.05 (3.42-8.03), CG = 2.83 (2.03-3.3), p = 0.015, ES = 0.541**       - **PSEC (AS/NDS):** **TA = 6.31 (5.04-9.87), CG = 3.59 (3.03-5.17), p = 0.023, ES = 0.507**       - **PSEC (IS/DS):** **TA = 4.96 (4.37-8.11), CG = 2.7 (2.38-3.74), p = 0.005, ES = 0.608** - **Within-Group Comparisons (AS vs. IS in TA):**   - **LoS:** **EO activation was significantly lower on the AS compared to the IS (p = 0.037, ES = 0.661).**   - **Static Stance:** **Multifidus (MF) activation on the AS was significantly higher than on the IS under the PSEO condition (p = 0.022, ES = 0.725).** |
| Main results | 16 | (*a*) Give unadjusted estimates and, if applicable, confounder-adjusted estimates and their precision (eg, 95% confidence interval). Make clear which confounders were adjusted for and why they were included | 8-13 | **Unadjusted and Adjusted Estimates with Precision:**  **1. Unadjusted Estimates (Mann-Whitney U and Wilcoxon Signed-Rank Tests):**  Since this study used **non-parametric tests** (Mann-Whitney U for between-group comparisons and Wilcoxon Signed-Rank for within-group comparisons), results were reported as **medians and interquartile ranges (IQR)** rather than means and standard deviations. Effect sizes (ES) were calculated for the magnitude of differences.   - **Postural Control Outcomes (Unadjusted Estimates)**:   - **LoS (AS in TA vs. NDS in CG):** **p = 0.019, ES = 0.524**   - **Lateral Sway (TA vs. CG, PSEO):** **p = 0.023, ES = 0.507**   - **Lateral Sway (TA vs. CG, PSEC):** **p = 0.002, ES = 0.659** - **Trunk Muscle Activation Outcomes (Unadjusted Estimates):**   - **External Oblique (IS/DS, TA vs. CG, LoS):** **p = 0.023, ES = 0.507**   - **Longissimus Dorsi (IS/DS, TA vs. CG, NSEC):** **p = 0.007, ES = 0.592**   - **Longissimus Dorsi (IS/DS, TA vs. CG, NSEO):** **p = 0.035, ES = 0.473**   - **Longissimus Dorsi (IS/DS, TA vs. CG, PSEO):** **p = 0.015, ES = 0.541**   - **Longissimus Dorsi (AS/NDS, TA vs. CG, PSEC):** **p = 0.023, ES = 0.507**   - **Longissimus Dorsi (IS/DS, TA vs. CG, PSEC):** **p = 0.005, ES = 0.608** - **Within-Group Comparisons (Unadjusted Estimates):**   - **External Oblique (AS vs. IS in TA, LoS):** **p = 0.037, ES = 0.661**   - **Multifidus (AS vs. IS in TA, PSEO):** **p = 0.022, ES = 0.725**   Since non-parametric tests were used, **95% confidence intervals (CIs) were not calculated**, as these tests do not produce standard error estimates for means. Instead, **effect sizes (ES) were reported**, which indicate the strength of the observed differences.  **2. Confounder-Adjusted Estimates and Justification for Adjustments:**  Given the **small sample size (n=20)** and use of **non-parametric tests**, **multivariate regression analysis was not performed**, and formal confounder-adjusted estimates (e.g., adjusted odds ratios with confidence intervals) were not calculated. However, potential confounders were addressed through **matching and side-specific comparisons**:   - **Matched Confounders:**   - **Age, sex, BMI, and lower limb length** were **statistically similar between groups (p > 0.05)**, reducing their confounding effects. - **Side-Specific Matching:**   - The **amputated side (AS) in TA was compared to the non-dominant side (NDS) in CG**, while the **intact side (IS) in TA was compared to the dominant side (DS) in CG** to account for potential dominance-related differences in balance and muscle activation. - **Normalization of EMG Data:**   - **Muscle activation was expressed as %MVIC (Maximum Voluntary Isometric Contraction)** to ensure **comparability across participants** and minimize the impact of individual strength differences. |
|  |  | (*b*) Report category boundaries when continuous variables were categorized | 8-13 | **Category Boundaries for Continuous Variables:**  In this study, most variables were analyzed as **continuous data** using **non-parametric tests** (Mann-Whitney U and Wilcoxon Signed-Rank), meaning that categorization was generally avoided. However, some continuous variables were categorized for subgroup analyses and eligibility criteria.  **1. Eligibility Criteria Categories:**   - **Body Mass Index (BMI):**   - **Included:** BMI **≤ 30 kg/m²**   - **Excluded:** BMI **> 30 kg/m²** - **Prosthesis Use Duration (for subgroup analysis within TA group):**   - **Short-term users:** **<3 years**   - **Long-term users:** **≥3 years**   **2. Limb-Specific Data Categorization:**   - To ensure valid between-group comparisons, lower limb data were categorized as follows:   - **Transtibial Amputee Group (TA):**     - **Amputated Side (AS)**     - **Intact Side (IS)**   - **Control Group (CG):**     - **Dominant Side (DS)**     - **Non-Dominant Side (NDS)**   - **Matching:**     - **AS in TA compared to NDS in CG**     - **IS in TA compared to DS in CG**   **3. Postural Control and EMG Data Categorization:**   - **Postural Stability Conditions:**   - **Normal Stability Eyes Open (NSEO)**   - **Normal Stability Eyes Closed (NSEC)**   - **Perturbed Stability Eyes Open (PSEO)**   - **Perturbed Stability Eyes Closed (PSEC)** - **Electromyography (EMG) Normalization:**   - Muscle activation data were **expressed as %MVIC (Maximum Voluntary Isometric Contraction)** to ensure comparability across participants. |
|  |  | (*c*) If relevant, consider translating estimates of relative risk into absolute risk for a meaningful time period | 8-13 | **Absolute vs. Relative Risk Considerations:**  This study does not involve **longitudinal follow-up** or **event-based risk assessment** (e.g., incidence of falls, injury rates), so **relative risk (RR) or absolute risk (AR) calculations were not applicable**. |

Continued on next page

| Other analyses | 17 | Report other analyses done—eg analyses of subgroups and interactions, and sensitivity analyses | 8-13 | None |
| --- | --- | --- | --- | --- |
| **Discussion** | | | | |
| Key results | 18 | Summarise key results with reference to study objectives | 14-19 | **Summary of Key Results in Relation to Study Objectives:**  This study aimed to investigate **postural control and trunk muscle activation mechanisms** following **unilateral transtibial amputation** and their effects on balance. The findings demonstrate significant **postural control adaptations** and **neuromuscular compensatory strategies**, particularly in trunk muscle activation, among transtibial amputees (TA).  **1. Postural Control Adaptations:**   - **Limits of Stability (LoS):**   - **TA participants exhibited greater LoS distance on the amputated side (AS) compared to the non-dominant side (NDS) of controls (CG) (p = 0.019).**   - This suggests that amputees may adopt **a widened base of support** and shift weight toward the intact limb to compensate for postural instability. - **Postural Sway:**   - **TA exhibited significantly greater lateral postural sway compared to CG under perturbed surface conditions (PSEO: p = 0.023, PSEC: p = 0.002).**   - The **loss of somatosensory feedback below the knee** likely contributes to increased postural instability, leading to a **greater reliance on visual and trunk muscle compensation mechanisms**.   **2. Trunk Muscle Activation and Compensation Mechanisms:**   - **External Oblique (EO) Compensation:**   - **EO activation on the intact side (IS) was significantly greater than on the AS in TA (p = 0.037) and higher than in CG during LoS (p = 0.023).**   - This suggests that **EO muscles on the intact side compensate for CoG shifts**, facilitating controlled movement toward the amputated limb. - **Multifidus (MF) Overactivation on the Amputated Side:**   - **MF activation on the AS was significantly higher than on the IS in TA under PSEO (p = 0.022).**   - This indicates an **increased neuromuscular demand on deep spinal stabilizers**, likely due to **reduced proprioceptive input** from the prosthetic limb. - **Longissimus Dorsi (LD) Activation and Trunk Stiffening:**   - **LD activation on the IS in TA was higher than in CG across multiple conditions (NSEC: p = 0.007, NSEO: p = 0.035, PSEO: p = 0.015, PSEC: p = 0.005).**   - **LD activation on the AS was also significantly higher than in CG under PSEC (p = 0.023).**   - These findings suggest that **transtibial amputees rely on LD activation to stiffen the trunk and prevent excessive perturbations of the center of mass (CoM), particularly during balance-challenging conditions.**   - The **increased activation of LD on the intact side compared to the AS during PSEO (although not statistically significant) may reflect the need for greater trunk muscle support on the intact side to counterbalance hip torque deficits.**   **3. Clinical and Rehabilitation Implications:**   - **The findings emphasize the importance of targeted rehabilitation strategies** to address:   - **Postural instability due to increased lateral sway and reliance on compensatory muscle activation patterns.**   - **Neuromuscular demands on deep and superficial trunk muscles, particularly the MF and LD.**   - **The need for training interventions that promote automatic deep trunk muscle activation and reduce excessive reliance on compensatory mechanisms.** - **Interventions such as motor control exercises (e.g., side planks, Swiss ball movements) may enhance MF activation and reduce postural sway**, improving overall balance control and reducing the risk of falls.   **Conclusion:** This study highlights **significant postural control adaptations and compensatory trunk muscle activation strategies in transtibial amputees**. Increased **postural sway, altered muscle activation patterns, and trunk stiffening mechanisms** reflect biomechanical adaptations aimed at maintaining stability. The findings provide **critical insights for rehabilitation strategies**, emphasizing the need for **targeted interventions** to optimize **postural control, trunk muscle function, and fall prevention** in individuals with transtibial amputation. |
| Limitations | 19 | Discuss limitations of the study, taking into account sources of potential bias or imprecision. Discuss both direction and magnitude of any potential bias | 19 | **Study Limitations and Potential Sources of Bias:**  While this study provides valuable insights into **postural control and trunk muscle activation in unilateral transtibial amputees (TA)**, several limitations must be acknowledged. These limitations may introduce potential **bias or imprecision**, affecting the **generalizability, accuracy, and scope of the findings**.  **1. Generalizability and Sample-Specific Limitations:**   - **Population Restriction:**   - The findings are **limited to male individuals** with **unilateral transtibial amputation due to traumatic causes** who use prosthetic limbs.   - **Potential Bias:** This **excludes female participants and individuals with amputation due to vascular disease or congenital conditions**, limiting the **generalizability to other amputee populations**.   - **Direction of Bias:** The results may **overestimate balance performance** compared to vascular amputees, who typically exhibit **poorer postural control due to comorbidities and reduced muscle strength**.   **2. Electromyographic (EMG) Measurement Limitations:**   - **Composite EMG Recordings During Limits of Stability (LoS):**   - EMG activations were recorded as a **single measure across all movement directions**, without distinguishing individual muscle activity **for each specific direction of movement**.   - **Potential Bias:** This approach **may obscure directional differences in muscle activation patterns**, limiting the ability to identify **specific compensatory muscle strategies** during lateral, anterior, or posterior shifts.   - **Direction of Bias:** The results may **underestimate the complexity of trunk muscle adaptations** in TA by failing to **capture directional muscle activation variability**.   - **Magnitude of Bias:** Moderate - while the composite measure provides valuable insights, a more detailed directional analysis could refine understanding of **muscle coordination strategies**.   **3. Standardization of Prosthetic Components and Suspension Systems:**   - **Potential Variability in Suspension Systems:**   - While **socket type was standardized**, **variability in suspension systems (active vs. passive vacuum) was present**.   - **Potential Bias:** Differences in suspension mechanisms **may influence weight distribution, proprioception, and postural control**, introducing variability in balance performance.   - **Direction of Bias:** The inclusion of both active and passive vacuum users **may increase variability in the results**, but the use of identical **carbon prosthetic feet** likely mitigates some inconsistencies.   - **Magnitude of Bias:** Low - because **prosthetic foot type was controlled**, ensuring that **differences in energy return and mechanical properties** did not confound results.   **4. Sample Size and Statistical Power:**   - **Small Sample Size (n=20):**   - The study included **only 10 transtibial amputees and 10 healthy controls**, limiting **statistical power** and increasing the risk of **Type II errors (failing to detect real differences)**.   - **Potential Bias:** With a larger sample, **some non-significant findings (e.g., certain EMG differences) might have reached statistical significance**.   - **Direction of Bias:** The results may **underestimate the true extent of postural and muscle activation differences** due to sample variability.   - **Magnitude of Bias:** Moderate - the small sample size affects statistical robustness but does not invalidate significant findings, which align with previous research.   **5. Cross-Sectional Study Design:**   - **Lack of Longitudinal Follow-Up:**   - The study design captures **a single time point**, preventing analysis of **progressive balance adaptations, long-term compensatory mechanisms, or rehabilitation effects**.   - **Potential Bias:** Differences in prosthesis experience and rehabilitation history were **not tracked over time**, meaning **findings may not represent ongoing neuromuscular adaptations**.   - **Direction of Bias:** The results **may not fully reflect the dynamic nature of postural control changes**, potentially **underestimating or overestimating** how **neuromuscular compensation evolves over time**.   - **Magnitude of Bias:** High - future **longitudinal studies are needed** to assess **how balance and muscle activation change with prolonged prosthesis use and rehabilitation**. |
| Interpretation | 20 | Give a cautious overall interpretation of results considering objectives, limitations, multiplicity of analyses, results from similar studies, and other relevant evidence | 14-20 | **Cautious Interpretation of Results Considering Study Objectives, Limitations, and Existing Evidence:**  This study aimed to investigate **postural control and trunk muscle activation mechanisms** in **unilateral transtibial amputees (TA)** and their implications for balance regulation. The findings highlight **significant postural control adaptations**, **neuromuscular compensatory strategies**, and **increased trunk muscle activation in TA**, particularly under **perturbed balance conditions**.  **1. Alignment with Study Objectives and Key Findings:**   - The study confirmed **greater postural instability in TA**, evidenced by **increased lateral sway under perturbed conditions (PSEO: p = 0.023, PSEC: p = 0.002)** and **greater reliance on the intact limb during postural shifts (LoS AS > NDS, p = 0.019)**. - **Increased activation of key trunk muscles, particularly the external oblique (EO), multifidus (MF), and longissimus dorsi (LD), was observed in TA compared to CG**, indicating **neuromuscular adaptations aimed at stabilizing balance**. - These findings suggest **compensatory trunk muscle recruitment strategies** to offset **somatosensory deficits, reduced mechanical stability, and altered weight distribution due to amputation**.   **2. Consideration of Study Limitations and Potential Bias:**  While the findings provide valuable insights, they should be interpreted **with caution** due to several **methodological constraints**:   - **Generalizability is limited** to **male traumatic amputees**, restricting applicability to **females or individuals with vascular amputations**, who may exhibit **greater postural deficits due to underlying comorbidities**. - **EMG analysis recorded composite activation** across all movement directions, **limiting insight into specific movement-related muscle activation patterns**. - **Small sample size (n=20) reduces statistical power**, potentially **underestimating some effects**, particularly in **within-group comparisons**. - **Cross-sectional design prevents long-term adaptation analysis**, meaning **the observed compensatory patterns may change with prosthesis experience and rehabilitation**.   **3. Multiplicity of Analyses and Statistical Considerations:**  The study performed multiple **between-group and within-group comparisons**, increasing the potential for **Type I errors (false positives)**. However, the **consistency of findings across multiple conditions and alignment with prior research** suggest that the observed effects are **biologically relevant and not due to random variation**.  **4. Comparison with Existing Literature and Theoretical Implications:**   - **Postural Sway and Balance Impairments:**   - Consistent with previous studies (**Hermodsson et al., Isakov et al., Fernie et al.**), this study found **greater postural sway in TA compared to CG, particularly under eyes-closed conditions**, suggesting **increased reliance on vision to compensate for reduced somatosensory input**.   - Similar to prior research, **amputees exhibited greater LoS on the amputated side**, likely due to **a widened base of support and weight-shifting adaptations**. - **Trunk Muscle Adaptations:**   - **The increased EO activation on the intact side in TA aligns with previous findings**, suggesting that **EO plays a key role in lateral stability and postural adjustments** during CoG shifts.   - **Higher MF and LD activation on the amputated side** suggests **greater neuromuscular effort to stabilize posture**, consistent with findings from **Sions et al. and Hendershot et al.**, which highlight **altered trunk stabilization strategies in lower limb amputees**.   **5. Clinical and Rehabilitation Implications:**  The findings underscore the **importance of targeted rehabilitation approaches** to:   - **Enhance postural stability by reducing excessive lateral sway** through **balance training and sensory reweighting exercises**. - **Address neuromuscular compensations**, particularly **overactivation of EO, MF, and LD muscles**, to optimize **trunk control and minimize fatigue-related instability**. - **Develop individualized interventions** incorporating **motor control exercises (e.g., side planks, Swiss ball movements)** to **train deep stabilizing muscles** and reduce reliance on compensatory mechanisms. |
| Generalisability | 21 | Discuss the generalisability (external validity) of the study results | 14-20 | **Generalisability (External Validity) of the Study Results:**  The external validity of this study refers to the extent to which its findings can be applied to broader populations beyond the study sample. While the results provide valuable insights into **postural control and trunk muscle activation in transtibial amputees (TA)**, several factors influence **how generalizable these findings are to other groups**.  **1. Strengths Enhancing Generalisability:**  **a) Well-Defined Inclusion Criteria:**   - Participants were carefully selected based on **clear inclusion/exclusion criteria**, ensuring a **homogeneous sample** of **active prosthetic users with no confounding musculoskeletal or neurological conditions**. - This **reduces variability within the study group**, allowing for a **clearer interpretation of amputation-specific postural adaptations**.   **b) Standardization of Prosthetic Components:**   - **All TA participants used total contact sockets and carbon prosthetic feet**, minimizing **variability in prosthesis type** that could affect postural control. - While suspension systems differed (active vs. passive vacuum), this **likely had minimal impact on balance outcomes**, as prosthetic feet play a more dominant role in postural stability.   **2. Factors Limiting Generalisability:**  **a) Sample Characteristics (Sex, Amputation Cause, Activity Level):**   - **Restricted to Male Participants:**   - The study exclusively included **male transtibial amputees**, meaning **results may not fully apply to female amputees**.   - **Sex-related differences in muscle mass, movement strategies, and balance control** suggest that females may **exhibit different neuromuscular adaptations**. - **Only Traumatic Amputees Were Included:**   - Individuals with **vascular or diabetic-related amputations were excluded**, limiting the applicability to **older or medically complex amputee populations**.   - **Vascular amputees typically exhibit poorer balance due to comorbidities such as neuropathy and reduced muscle strength**, meaning **this study likely overestimates balance performance in the general transtibial amputee population**. - **Participants Were High-Functioning Prosthetic Users (K3-K4 Levels):**   - All TA participants had a **Medicare Functional Classification Level of K3 or K4**, indicating **moderate to high activity levels**.   - Results may **not be applicable to lower-functioning amputees (K1-K2),** who may exhibit **greater postural instability and weaker compensatory mechanisms**.   **b) Small Sample Size:**   - The study included **only 10 transtibial amputees and 10 controls**, which is a **small sample size** for drawing strong conclusions about **broader amputee populations**. - **Larger, more diverse samples** are needed to **improve statistical power** and confirm whether the observed findings are **consistent across different subgroups**.   **c) Cross-Sectional Study Design:**   - Since this was a **cross-sectional study**, results **only reflect postural control at a single time point**. - **Longitudinal studies are needed** to determine **how postural control changes over time** and whether **rehabilitation, prosthetic use duration, or aging influence balance adaptations**.   **d) Laboratory-Based Assessments vs. Real-World Conditions:**   - **All assessments were conducted in a controlled, distraction-free laboratory environment**, which may not fully reflect **real-world postural challenges** (e.g., walking on uneven terrain, navigating obstacles). - **Amputees often encounter additional environmental and cognitive demands** in daily life, meaning real-world balance performance **may be worse than observed in the study**.   **3. Implications for Broader Populations:**   - **Findings are most applicable to**:   - **Male, high-functioning, traumatic transtibial amputees**   - **Individuals using total contact sockets and carbon prosthetic feet**   - **Active prosthesis users (K3-K4 level)** - **Findings may not be fully generalizable to**:   - **Female amputees** (potential sex-related differences in muscle function and balance control)   - **Older or vascular amputees** (higher fall risk due to comorbidities)   - **Lower-functioning prosthetic users (K1-K2)**   - **Amputees using different prosthetic foot technologies or socket systems**   - **Real-world settings, where additional balance challenges exist**   **As aresult,** while the study provides **valuable insights into postural control adaptations in transtibial amputees**, its findings should be interpreted **with caution when applied to different amputee populations**. **Future research should include larger, more diverse samples, female participants, vascular amputees, and longitudinal designs** to improve the **generalizability and clinical applicability of these findings**. |
| **Other information** | |  | | |
| Funding | 22 | Give the source of funding and the role of the funders for the present study and, if applicable, for the original study on which the present article is based | 20 | No funding was received. |

*Give information separately for cases and controls in case-control studies and, if applicable, for exposed and unexposed groups in cohort and cross-sectional studies.

**Note:** An Explanation and Elaboration article discusses each checklist item and gives methodological background and published examples of transparent reporting. The STROBE checklist is best used in conjunction with this article (freely available on the Web sites of PLoS Medicine at http://www.plosmedicine.org/, Annals of Internal Medicine at http://www.annals.org/, and Epidemiology at http://www.epidem.com/). Information on the STROBE Initiative is available at [www.strobe-statement.org](http://www.strobe-statement.org).
